# Organoids and metastatic orthotopic mouse model for mismatch repair-deficient colorectal cancer

**DOI:** 10.3389/fonc.2023.1223915

**Published:** 2023-09-08

**Authors:** Yurong Song, Travis D. Kerr, Chelsea Sanders, Lisheng Dai, Shaneen S. Baxter, Brandon Somerville, Ryan N. Baugher, Stephanie D. Mellott, Todd B. Young, Heidi E. Lawhorn, Teri M. Plona, Bingfang Xu, Lei Wei, Qiang Hu, Song Liu, Alan Hutson, Baktiar Karim, Sandra Burkett, Simone Difilippantonio, Ligia Pinto, Johannes Gebert, Matthias Kloor, Steven M. Lipkin, Shizuko Sei, Robert H. Shoemaker

**Affiliations:** ^1^ Frederick National Laboratory for Cancer Research, Vaccine, Immunity, and Cancer Directorate, Frederick, MD, United States; ^2^ Frederick National Laboratory for Cancer Research, Laboratory Animal Sciences Program, Frederick, MD, United States; ^3^ Frederick National Laboratory for Cancer Research, Clinical Laboratory Improvement Amendments (CLIA) Molecular Diagnostics Laboratory, Frederick, MD, United States; ^4^ Frederick National Laboratory for Cancer Research, Genomics Laboratory, Frederick, MD, United States; ^5^ Department of Biostatistics and Bioinformatics, Roswell Park Comprehensive Cancer Center, Buffalo, NY, United States; ^6^ Molecular Histopathology Laboratory, Frederick National Laboratory for Cancer Research, Frederick, MD, United States; ^7^ Molecular Cytogenetics Core Facility, National Cancer Institute, Frederick, MD, United States; ^8^ Department of Applied Tumor Biology, Institute of Pathology, University of Heidelberg, Heidelberg, Germany; ^9^ Department of Medicine, Weill Cornell Medical College, Cornell University, New York, NY, United States; ^10^ Chemopreventive Agent Development Research Group, Division of Cancer Prevention, National Cancer Institute, Bethesda, MD, United States

**Keywords:** mismatch repair deficiency, Lynch syndrome, microsatellite instability, chromosome instability, MSH2, organoid, mouse model, colorectal cancer

## Abstract

**Background:**

Genome integrity is essential for the survival of an organism. DNA mismatch repair (MMR) genes (e.g., *MLH1, MSH2, MSH6*, and *PMS2*) play a critical role in the DNA damage response pathway for genome integrity maintenance. Germline mutations of MMR genes can lead to Lynch syndrome or constitutional mismatch repair deficiency syndrome, resulting in an increased lifetime risk of developing cancer characterized by high microsatellite instability (MSI-H) and high mutation burden. Although immunotherapy has been approved for MMR-deficient (MMRd) cancer patients, the overall response rate needs to be improved and other management options are needed.

**Methods:**

To better understand the biology of MMRd cancers, elucidate the resistance mechanisms to immune modulation, and develop vaccines and therapeutic testing platforms for this high-risk population, we generated organoids and an orthotopic mouse model from intestine tumors developed in a Msh2-deficient mouse model, and followed with a detailed characterization.

**Results:**

The organoids were shown to be of epithelial origin with stem cell features, to have a high frameshift mutation frequency with MSI-H and chromosome instability, and intra- and inter-tumor heterogeneity. An orthotopic model using intra-cecal implantation of tumor fragments derived from organoids showed progressive tumor growth, resulting in the development of adenocarcinomas mixed with mucinous features and distant metastasis in liver and lymph node.

**Conclusions:**

The established organoids with characteristics of MSI-H cancers can be used to study MMRd cancer biology. The orthotopic model, with its distant metastasis and expressing frameshift peptides, is suitable for evaluating the efficacy of neoantigen-based vaccines or anticancer drugs in combination with other therapies.

## 1 Introduction

Genome integrity is maintained by multiple pathways. Among these, the accuracy of DNA replication relies on DNA mismatch repair (MMR) genes, including *MLH1, MSH2, MSH6*, and *PMS2*. Germline deficiency in MMR genes (MMRd) leads to Lynch syndrome (LS) via monoallelic mutation or constitutional mismatch repair deficiency syndrome (CMMRD) via biallelic mutation. Individuals with MMRd usually have a high mutation burden due to an inability to repair errors that occur during DNA replication, especially in coding microsatellite regions. The accumulation of these mutations in the genome leads to a genomic state of high microsatellite instability (MSI-H). Thus, carriers with pathogenic MMR variants usually have an increased lifetime risk for developing various cancers with early cancer onset (e.g., colorectal cancer [CRC] or hereditary non-polyposis colorectal cancer [HNPCC], endometrial cancer, gastric cancer, and ovarian cancer mainly for LS, and brain cancer, gastrointestinal cancer, and lymphomas for CMMRD) (1, 2). In addition, somatic mutations in MMR genes can also lead to MSI-H tumors (3–5). The disease penetrance and age of onset vary among the four deficient MMR genes. *MLH1* and *MSH2* mutations are mainly implicated in LS, while *PMS2* and *MSH6* mutations are predominantly responsible for CMMRD. The incomplete disease penetrance suggests that other genetic or epigenetic factors are also involved in the disease etiology.

Recent advances in immuno-oncology have supported tremendous progress in the treatment of MMRd/MSI-H cancers by engaging patients’ own immune system. To date, FDA has approved three immune checkpoint inhibitors for treatment of MSI-H metastatic CRC (6–8). In studies using the anti-PD-1 antibody pembrolizumab in heavily pretreated MMRd/MSI-H patients, the overall response rate was 40% and 71% in MSI-H CRC and non-CRC cancers, respectively (9), while a 53% objective response rate was observed across tumor types (10). In a pooled phase 2 (KEYNOTE-158, KEYNOTE-164, and KEYNOTE-051) trials analysis, the objective response rate was 33.3%. Treatment with another anti-PD-1 antibody, nivolumab, showed a similar objective response rate (31.3%) in chemotherapy-refractory MMRd/MSI-H CRC patients, with 14.3 months of median progression-free survival. The most recent phase 3 study showed an objective response of 45% with pembrolizumab used as a first-line therapy in MMRd/MSI-H/metastatic CRC patients, vs. 33% with chemotherapy (11). The combination therapy using nivolumab and the anti-CTLA4 antibody ipilimumab showed an increased overall response rate of 55% in advanced MMRd/MSI-H CRC patients (12). Responders had marked expansion of T cells targeting frameshift neopeptides (10), which contributes to overall disease control and tumor suppression. These frameshift neoantigens have been the basis for immune-based therapies and have been actively explored as preventative cancer vaccine antigens for individuals at risk for MMRd/MSI-H cancers (13–16). With the best overall response rate of 55% in advanced MMRd/MSI-H CRC patients (12), a model system that recapitulates human disease is urgently needed to better understand the disease biology and heterogeneity, elucidate resistance mechanisms, and test new treatment strategies and preventive approaches preclinically.

Established cell lines, organoids, and patient-derived xenograft (PDX) models from LS CRC patients are of great value to the research community. However, they cannot be used to develop a mouse model for immunotherapy testing due to the immunodeficiency in NSG mice. Organoids and orthotopic models derived from MMR proficient tumors from genetically engineered mouse models (GEMMs) (e.g., *Apc^Min^* model) are great tools for CRC studies. However, there have not been organoid-based models for MMRd/MSI-H intestinal tumors. While GEMMs for MMRd/MSI-H CRC have been generated and widely used in cancer biology and therapeutic treatment studies (17, 18), intestinal tumor development in these models usually takes many months (19). To accelerate the discovery process for this high-risk population and provide a feasible model system for *in vitro* and *in vivo* screening of vaccine and therapeutic candidates, we aimed to generate organoids and develop an orthotopic mouse model for MMRd/MSI-H CRC. The *Msh2^LoxP/LoxP^*;*Villin-Cre* (*VCMsh2)* mouse model is particularly useful because these mice are predisposed to develop intestine tumors (mainly from the small intestine) without metastasis (19). We generated organoids from small intestine and colon tumors from this model and developed an intra-cecal implantation model using organoid-derived tumor fragments. The established tumor organoids, driven by Msh2-deficiency, can be used to better understand MMRd/MSI-H cancer biology and tumor heterogeneity. The orthotopic model, with distant metastasis and frameshift (FS) neoantigen expression, may be suitable for testing neoantigen-based vaccines and developing new approaches for effective combination treatments.

## 2 Materials and methods

### 2.1 Mice

*VCMsh2* mice (19) were provided by Dr. Winfried Edelmann at Albert Einstein College of Medicine and maintained individually by crossing to C57BL/6J (the Jackson Laboratory) mice. The experimental mice were generated by crossing *Msh2^LoxP/+^;Villin-Cre* to *Msh2^LoxP/LoxP^* mice. Animals were fed *ad libitum* on a normal chow diet (Purina 5L79 – regular). Genotyping was performed as described previously (19). C57BL/6J mice and NOD SCID gamma (NSG™) mice (the Jackson Laboratory) were used as recipients for *in vivo* tumorigenicity studies.

NCI Frederick is accredited by AAALAC International and follows the Public Health Service Policy for the Care and Use of Laboratory Animals. Animal care was provided in accordance with the procedures outlined in the “Guide for Care and Use of Laboratory Animals” (National Research Council, 1996; National Academy Press; Washington, D.C.).

### 2.2 Tumor and wildtype organoid generation

Tumor organoids were generated from tumor-bearing *VCMsh2* mice following published protocols (20, 21). Briefly, tumors were excised and sliced into small pieces from tumor periphery. Tumor pieces were not assessed for tumor cell content for organoid generation. They were incubated in EDTA chelation buffer for 1 hour on ice, then digestion buffer for 2 hours at 37°C. After washing with cold PBS, supernatant was collected by allowing tumor fragments to settle under normal gravity for 1 minute, then single cells in the supernatant were pelleted and washed with PBS. Cells were resuspended in growth factor-reduced and phenol red-free Matrigel (Corning #356231) on ice and plated in 24-well plates at 15,000 cells/50 µL. The plate was then incubated at 37°C to polymerize the Matrigel. After 15 minutes, 500 µL basal culture medium (BCM; **Supplementary Material**) containing 50 ng/mL murine EGF was added to each well, which was refreshed every 2–3 days. WT organoids were generated from a WT littermate (*Msh2^+/+^;Villin-Cre^+/+^*) following a modified protocol (22, 23). Briefly, intestinal villi were gently scraped off and discarded, and the tissue was cut into 1-cm pieces and incubated in a 50 mL Falcon tube containing PBS with 5 mM EDTA for 45 minutes at 4°C in a HulaMixer set to 30 rpm for orbital rotation with a 60° turning angle for reciprocal rotation. After vigorously shaking by hand, tissue fragments were collected on a sieve and discarded. The flow-through was then centrifuged and the supernatant was washed by adding cold RPMI 1640 (Gibco). After centrifugation, the resulting pellet containing detached crypts was washed and resuspended in RPMI 1640, then purified by filtration through 70 µm mesh. The resulting pellet was resuspended in complete culture medium (CCM; **Supplementary Material**). To each well of a 24-well plate, 100 µL of crypts with 40 µL of additional CCM and 60 µL of Matrigel was added to a pre-warmed 24-well plate. After polymerizing the Matrigel, 300 µL of prewarmed CCM was added to each well. Organoid growth was monitored daily using an inverted microscope. CCM was gently pipetted off and fresh CCM with fresh Matrigel was replenished every 2 days. For passaging, organoids and Matrigel were mechanically disrupted using a P1000 pipet tip and collected and washed with BCM or CCM, then resuspended and plated at 50 µL Matrigel/well. To freeze in liquid nitrogen, organoids were disrupted using a P1000 pipette with cut-off tips and transferred into a 15 mL Falcon tube, then washed with 5 mL of BCM or CCM and centrifuged at 200 × *g* for 2 minutes. The pellet was resuspended in BCM with 20% fetal bovine serum (FBS) and 10% dimethyl sulfoxide (DMSO). Aliquots of 1 mL per cryovial were placed in a Corning CoolCell^®^ container (Corning, NY) in −80°C freezer, then transferred to vapor-phase liquid nitrogen for long-term storage.

### 2.3 Organoid injection and tumor fragment implantation

Mechanically disrupted organoids and Matrigel were collected and pipetted up and down ten times to disassociate the organoids. After resuspending in 30 mL of PBS, organoids were further disassociated by pipetting ten times, and resuspended in 2 mL BCM containing 50% Matrigel after removing the supernatant and a majority of the Matrigel using a P1000 pipet. A small volume of organoid suspension was further digested with TrypLE at 37°C for 10 minutes to obtain a single-cell suspension, then FBS was added to stop the TrypLE and clumps were resuspended by pipetting up and down. Cells were counted using a hemocytometer under a microscope, then the number of cells in each volume was calculated for the original organoid suspension. The appropriate number of cells was diluted in a final volume containing 50% Matrigel for subcutaneous injection. Under anesthesia, organoids in 100 µL with 50% Matrigel were injected subcutaneously (s.c.) to gender-matched, syngeneic recipients by making a small incision at the injection site and injecting using 20G needles. Animals were monitored daily for tumor growth. Tumor volumes were measured using calipers and calculated using the formula L*W*H* π/6 [3D]. For serial passaging, s.c. organoid tumors were harvested using sterile technique once they reached 1 cm. Briefly, tumors were excised and pieces, which appeared to be solid (to avoid thick mucinous or cystic region), were dissected. To avoid the necrotic center of a tumor mass, tumor pieces from the periphery were sliced into 2x2-mm tumor fragments (approximately 30 mg each) and implanted s.c. into recipients. Tumor fragments were not assessed for tumor cell content. Fragments were also viably frozen in vapor-phase liquid nitrogen for implantation later. While holding the small incision with forceps in a tented fashion, a tumor fragment was placed gently into the right flank by pushing the fragment into the pocket. One or two drops of 0.25% Bupivacaine were applied to the incision site before closing the incision with one surgical staple.

Intra-cecal tumor fragment implantation was performed in syngeneic mice by following established procedures (24, 25). Briefly, the blind-ended pouch of the cecum was exteriorized and supported on a sterile pre-cut gauze after a small midline abdominal incision was made. Warmed sterile saline was used to keep the cecum moist. A figure-eight stitch was placed onto the cecum using absorbable suture material. A small, approximately 3x3-mm area of the cecal wall under the suture was lightly damaged by grasping and pulling with serrated forceps to facilitate the infiltration of cancer cells. A fresh tumor fragment generated from s.c. tumor tissue (see above) was positioned under the suture on the cecal wall. The stitch was then tied down. After returning the cecum to the abdominal cavity, the peritoneal layer was closed with a 5-0, absorbable suture and the skin incision was closed with 1–2 wound clips after applying 1–2 drops of 0.25% Bupivacaine to the incision. Wound clips were removed after 10–14 days. Animals were closely monitored during post-surgery recovery.

### 2.4 Histopathology and immunostaining

Tumors were removed and fixed in 10% neutral buffered formalin overnight, transferred to 70% ethanol, and then routinely processed and embedded in paraffin. Hematoxylin and eosin (H&E) and immunohistochemical (IHC) staining were carried out on 4-μm sections as previously described (26) (**Supplementary Table 1**). Slides were digitally imaged using an Aperio ScanScope Scanner (Leica Biosystems). Staining was quantified using a HALO^®^ image analysis platform (Indica labs, Albuquerque, NM).

### 2.5 Microsatellite instability detection

For MSI detection, primers were designed (**Supplementary Table 2**; Integrated DNA Technologies, Coralville, IA, USA) for mouse target regions of MSI markers (L24372, U12235, Bat64, Bat30, Bat37, Bat59, and Bat67) on mouse build GRCm38.p6 as reported previously (27–31). PCR amplification was carried out with the Platinum™ SuperFi™ PCR Master Mix using cycling conditions in **Supplementary Table 3**. Fragment analysis and Sanger sequencing were performed as previously described (31). Bat67 fragment data were validated by Sanger sequencing (31). WT tail and organoid DNA were used as controls. Positivity was scored when at least a 1-bp shift of the repeat was observed or if new peaks appeared that were not present in the control tissue.

### 2.6 Frameshift mutation detection

DNA from organoids was extracted using the Qiagen DNeasy Blood & Tissue kit (Qiagen, Valencia, CA). Targeted sequencing was performed using the rhAmpSeq amplicon sequencing system (Integrated DNA Technologies IDT, Coralville, IA) with a dual-indexing strategy. Approximately 55 ng of purified DNA was combined with rhAmpSeq primers and cycled using the following conditions: 1 cycle of 95°C for 10 minutes, followed by 10 cycles of 95°C for 15 seconds and 61°C for 4 minutes, enzyme deactivation by 1 cycle of 99.5°C for 15 minutes and 4°C hold at the end. After bead-based cleanup using AMPure XP beads (Beckman Coulter, Brea, CA), amplicons were combined with IDT indexes for Illumina sequencing. PCR was performed using the following index PCR conditions: 1 cycle of 95°C for 3 minutes, followed by 18 cycles of 95°C for 15 seconds, 60°C for 30 seconds, and 72°C for 30 seconds; extension by 1 cycle of 72°C for 1 minute and 4C hold at the end. After a second bead-based cleanup, amplicon libraries were sized and quantified using a D1000 ScreenTape on TapeStation (Agilent Technologies, Santa Clara, CA) and a KAPA q-PCR system (Roche, Frederick, MD). Sequencing libraries were diluted to 4 nM, combined, and denatured for loading into the sequencing system. The final loading concentration was 12 pM. Sequencing was performed on an Illumina MiSeq system (Illumina, San Diego, CA) using a V3 reagent kit with a 1 x 151 bp length for the amplicon and 2 x 8 bp for the indexes.

For data analysis, index primers on raw reads from FASTQ files were trimmed and aligned to mouse reference genome Mus_musculus.GRCm39.dna.toplevel.fa using in-house-developed pipeline (available upon request). For each target FSM detection, the genomic site in all samples was revisited to extract the reads supporting the mutant or WT allele. The numbers of mutant and WT reads were used to calculate the insertion/deletion (indel) variant allele frequency (VAF) across all samples. To distinguish mutations from background errors, each indel’s background error rate distributions were modeled by fitting its VAF from all WT control samples into a Weibull distribution, then each tumor sample’s VAF was compared to the fitted distribution as previously described (32). A sample was defined as positive when the sample’s VAF of a mutation was significantly above background (*p* < 0.05, after multiple testing correction using the false discovery rate [FDR] method).

### 2.7 *Ctnnb1*, *Apc*, and *Trp53* mutation detection

For *Ctnnb1* mutation detection, 18 sets of Sanger sequencing primers were designed to cover all coding exons (**Supplementary Table 4**). For *Apc* mutation detection, seven hotspot mutations reported in *VCMsh2* tumors (19) were assessed by fragment analysis or Sanger sequencing as described previously (31). Briefly, for fragment analysis, all primers were ordered (**Supplementary Table 2**; Integrated DNA Technologies) and amplified on ProFlex PCR System (Thermo Fisher Scientific, Waltham, MA) using PCR conditions stated in **Supplementary Table 3**. The resulting products were then checked for quality and concentration with Agilent’s 2100 Bioanalyzer (Agilent Technologies, Santa Clara, CA) and Agilent’s DNA 1000 kit, using Bioanalyzer software version 2100 Expert B.02.11 SI824. Amplified samples were diluted with molecular grade water up to a 1:15 ratio as needed. A master mix combining Hi-Di™ Formamide (8.5 µL per reaction; Thermo Fisher Scientific), GeneScan™ 500 LIZ™ dye size standard (0.5 µL per reaction; Thermo Fisher Scientific), and 1 µL of diluted sample was created and incubated at 95°C for 5 minutes followed by 4°C for 2 minutes in the ProFlex™ PCR System. Samples were then processed on 3730xl DNA Analyzer (Thermo Fisher Scientific), using 96 capillary 50 cm array, a DS-33 Matrix Standard Kit (Dye Set 5; Thermo Fisher Scientific), and 3730XL Data Collection Software (version 5.0; Thermo Fisher Scientific). Data were then analyzed and overlayed with GeneMapper™ software (version 5.0; Thermo Fisher Scientific). Length of the PCR product in the testing sample was compared to the WT sample and the altered length was defined as instability. MSI status was classified as MSI-high (MSI-H) (instability at 2 or more microsatellite loci) and microsatellite stable (MSS) (instability at 1 locus or none).

For Sanger sequencing, primers were designed and amplified (**Supplementary Tables 2**, **3**). PCR products were purified using exonuclease I (GE Healthcare, Pittsburgh, PA) and shrimp alkaline phosphatase (SAP; Affymetrix USB) by incubating the sample mixture at 37°C for 15 minutes, then 80°C for 15 minutes, followed by a 4°C hold in the ProFlex™ PCR System. The purified amplicon then proceeded into cycle sequencing with BigDye™ Terminator v3.1 Cycle Sequencing Kit and M13 forward and M13 reverse primers (Thermo Fisher Scientific) using the following conditions in the ProFlex™ PCR System: 96°C for 1 minute, 25 cycles of 96°C for 10 seconds, 50°C for 5 seconds, 60°C for 1 minute and 15 seconds; followed by a hold at 4°C. Samples were then processed on 3730xl DNA Analyzer using 96 capillary 50cm array, Sequencing Standards, BigDye™ Terminator v3.1 Kit (Thermo Fisher Scientific), and 3730XL Data Collection Software. Data were then analyzed using Mutation Surveyor (version 5.1.2; SoftGenetics, State College, PA).

For *Trp53* mutation detection, 10 sets of PCR reactions were run for each sample using the primers described in **Supplementary Table 5**. *Trp53*-coding exon regions and splicing junctions were amplified using the following conditions: 95°C for 2 minutes, 36 cycles of 94°C for 15 seconds, 58°C for 15 seconds, and 68°C for 1 minute; and a hold at 68°C for 1 minute. After quantifying using TapeStation, amplicons from each sample were pooled by equal molarity and barcoded using the following conditions: 95°C for 2 minutes, 15 cycles of 94°C for 15 seconds, 56°C for 15 seconds, and 68°C for 1 minute, and a hold at 68°C for 1 minute. Barcoded PCR products were treated with Exonuclease I (New England Biolabs, Ipswich, MA) at 37°C for 15 minutes and purified using the Ampure XP protocol (Thermo Fisher Scientific). Purified amplicons were pooled together based on equal molarity, then pooled libraries were quantified using TapeStation and the final concentration was determined using a Qubit assay (Thermo Fisher Scientific). Paired-end sequencing was performed on an Illumina MiSeq sequencer using MiSeq V2 reagent kits 500-cycles (Illumina). The sequencing quality was monitored and the raw FASTQ files were downloaded for data analysis. Paired-end reads were trimmed using Trimmomatic (33). Top 100-300 most common reads for each sample were identified and sequence reads containing mutations with a frequency greater than 4% were detected, and the mutation frequency was calculated using custom-developed Python scripts. The sequence reads with mutations were visualized by aligning them with the WT mouse *Trp53* sequence using SnapGene software (Insightful Science; available at snapgene.com).

### 2.8 Spectral karyotyping

To evaluate chromosome instability (CIN), SKY analysis was performed as described previously (31). Briefly, organoids were disassociated from Matrigel by incubating with Dispase II, and single-cell suspensions were generated by incubating with TrypLE (ThermoFisher Scientific). Cells were then arrested in metaphase by incubating with Colcemid™ (10 µg/mL; 15210-040, KaryoMAX ® Colcemid Solution, Invitrogen, Carlsbad, CA) for 3 hours, treated with hypotonic solution (KCl 0.075M, 6858-04, Macron Chemical) for 15 minutes at 37°C, and then fixed with methanol:acetic acid 3:1. The prepared metaphase spread slides were aged overnight and then hybridized with the 21-color mouse SKY paint kit (FPRPR0030, ASI) in a humidity chamber at 37°C for 16 hours (34). Spectral images were acquired using a Hyper Spectral Imaging System (Applied Spectral Imaging Inc., CA) mounted on top of an epi-fluorescence microscope (Imager Z2, Zeiss) and analyzed using HiSKY 7.2 acquisition software (GenASIs, Applied Spectral Imaging Inc., CA). An average of 10–15 mitoses of comparable staining intensity and quality were examined per organoid line and evaluated for chromosomal abnormalities.

### 2.9 Multiplex *in situ* hybridization staining

The expression of WT Lgr5 and WT and mutant Asxl1 were detected by staining 5 μm formalin-fixed, paraffin-embedded (FFPE) tissue sections with the following probes: Mm-Lgr5-O2-C1 (ACD, Cat# 1237631-C1), RNAscope® 2.5 LS Probes Mm-Asxl1-C1 (ACD, Cat# 421968), and Mm-Asxl1-O1-C2 (ACD, Cat# 1149108-C2), and analyzing with the LS Multiplex Fluorescent Assay (ACD, Cat# 322800) using the Bond RX autostainer (Leica Biosystems). Tissue sections were pretreated with Bond Epitope Retrieval Solution 2 (Leica Biosystems) at 95°C for 15 minutes, Protease III (ACD) at 40°C for 15 minutes, and then a 1:750 dilution of TSA-Cyanine 3 Plus and TSA-Cyanine 5 Plus (Akoya Biosciences). The RNAscope® 3-plex LS multiplex negative control probe (*Bacillus subtilis* dihydrodipicolinate reductase (*dapB*) gene in channels C1, C2, and C3, Cat# 320878) and the RNAscope® LS 2.5 3-plex positive control probe-Hs were used as controls. Slides were digitally imaged using an Aperio ScanScope FL Scanner (Leica Biosystems). Images were analyzed and quantified using a HALO® image analysis platform (Indica labs, Albuquerque, NM).

### 2.10 Statistical analysis

All graphs and statistical analyses were made using GraphPad Prism 9 (GraphPad Software, San Diego, CA). All statistical tests were two-sided and *p* < 0.05 was considered significant unless otherwise stated. Kaplan–Meier survival curves were plotted, and the log-rank test was used for the median overall survival comparisons.

## 3 Results

### 3.1 Organoid generation and characterization by markers

**Figure 1:**
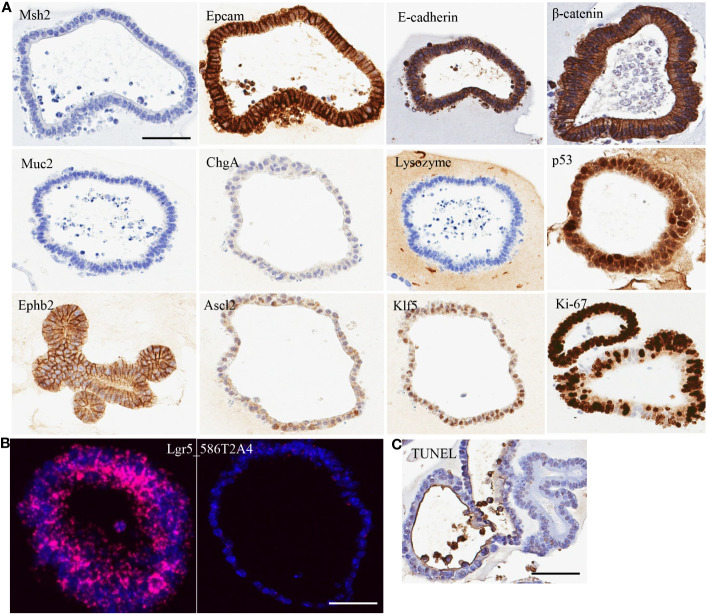
Characterization of organoid line 586T2A4 by **(A)** IHC staining, **(B)** mRNA *in situ* hybridization, and **(C)** TUNEL staining. Scale bar: 50 µm.

Organoids were successfully generated from small intestine and colon tumors developed in *VCMsh2* mice with 59% and 100% success rate at the animal level, respectively (**Supplementary Table 6**). Cells seeded in one well were treated as one organoid line. In total, 125 small intestine and 29 colon tumor organoid lines were established (passaged up to P3 *in vitro*) with some from the same tumor (20% and 57% success rate at the line level, respectively). One healthy organoid line from intestinal crypt cells from a WT littermate control was generated. Organoids were grown in Matrigel supplemented with EGF. Morphologically, most tumor organoids were very similar (round or round with small protrusions) except organoid line 586T2A4, which had convoluted, tube-like structures *in vitro* (**Supplementary Figure 1A**). H&E staining showed that these organoids were composed of either single or multiple cell layers with an open lumen filled with dead cells (**Supplementary Figure 1C**). To confirm the *Msh2* deletion by Villin-Cre *in vivo*, Msh2 IHC staining was performed on FFPE sections prepared from organoid pellets and *de novo VCMsh2* tumors. As expected, Msh2 was not expressed in *VCMsh2* tumors or organoids (**Figure 1A**; **Supplementary Figures 1D**, **2A**), while strong expression was detected in WT intestine (**Supplementary Figure 1D**).

To evaluate the cell type of the organoids, a panel of markers were assessed using IHC on FFPE sections made from 10 organoid lines. All organoids tested had very high expression of epithelial markers (e.g., Epcam and E-cadherin), but didn’t express Muc2 (goblet cell marker), chromogranin A (ChgA; enteroendocrine marker), or lysozyme (Paneth cell marker) (**Figure 1A**; **Supplementary Figure 2A**). One organoid line (961T3C3) had very low expression of Muc2, ChgA, and lysozyme. This indicates that the organoids were of epithelial cell origin. To determine if tumor organoid cells were of progenitor/stem cell origin, crypt cell markers Klf5, Ephb2, and Ascl2 expression were assessed using IHC, revealing high expression of these markers (**Figure 1A**; **Supplementary Figure 2A**). Lgr5 is a well-accepted intestine stem cell marker. Lgr5 expression was further assessed by mRNA *in situ* hybridization, showing strong but heterogenous positivity (**Figure 1B**; **Supplementary Figures 2B**, **3**). Some organoids showed high Lgr5 expression, but others low or no Lgr5 expression within the same organoid line. As expected, Ki67 IHC showed a high proliferation rate regardless of the Lgr5 expression level, along with a low apoptosis rate as assessed by the TUNEL assay (**Figure 1C**; **Supplementary Figure 2C**). These tumor organoids also showed strong membrane staining of β-catenin (**Figure 1A**; **Supplementary Figure 2A**). **Supplementary Table 7** summarizes the staining quantification used for **Figure 1**.

To determine if the antigen presentation complex was functional in tumor organoids, flow cytometry analysis of MHC class I and B2M was performed, revealing that the organoids had very low basal levels of H-2Db, H-2Kb, and B2M (**Supplementary Figure 4**). However, MHC class I expression could be induced by IFNγ treatment. H-2Db, H-2Kb, and B2M expression were significantly increased compared to non-treated controls, indicating that the MHC class I antigen processing and presentation machinery was functional in these tumor organoids.

### 3.2 Microsatellite and chromosome stability

**Figure 2:**
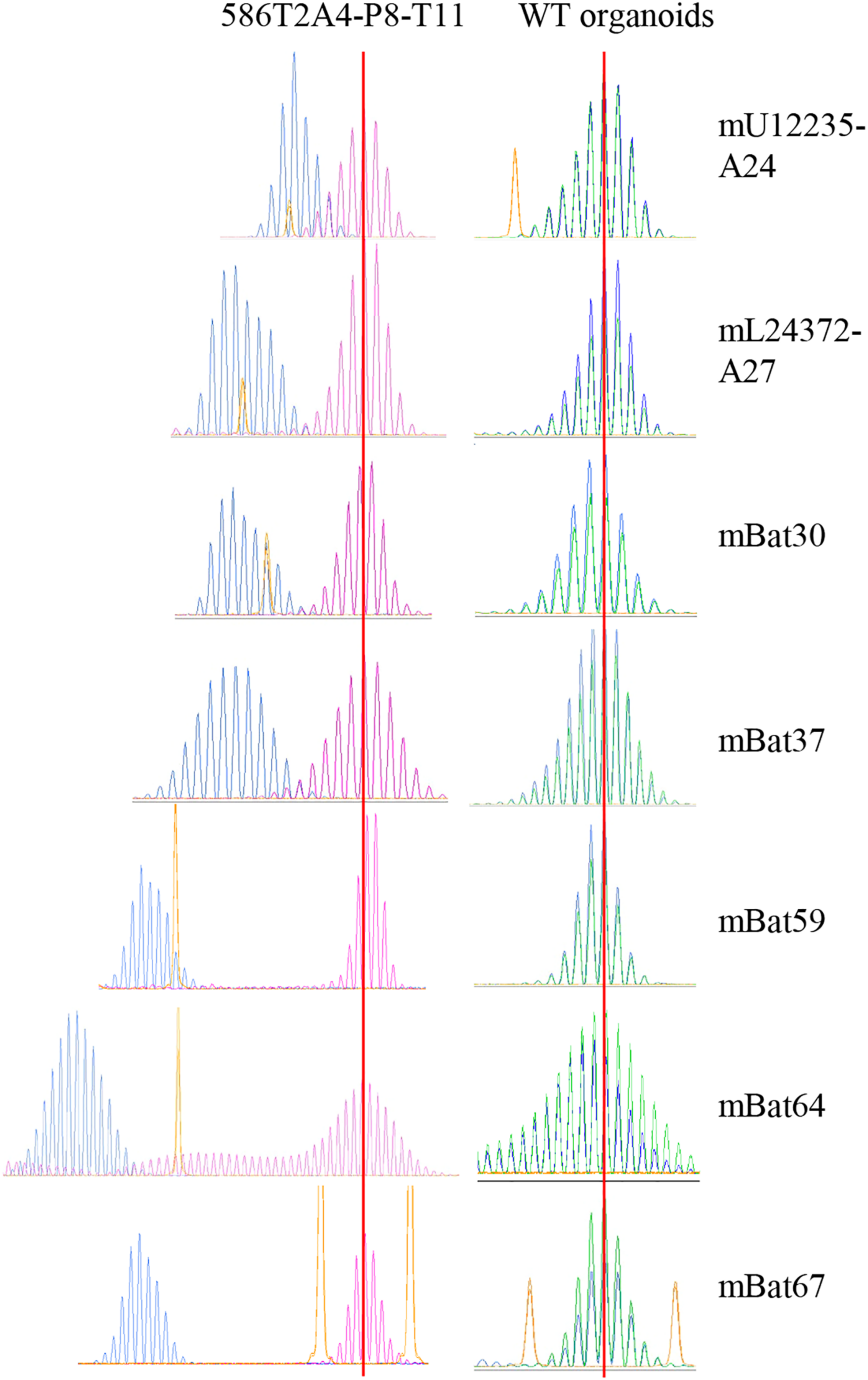
Fragment analysis of seven MSI markers in 586T2A4-P8-T11 and WT organoids. Blue peaks are 586T2A4-P8-T11 and WT organoids, pink or green peaks are WT tail, and yellow peaks are size markers. Two vertical red lines align the WT fragment peaks in WT tail.

MMRd cells characteristically show MSI-H. To confirm the MSI status in the tumor organoids, up to seven MSI markers were assessed in 27 tumor organoids and WT organoids via DNA fragment length analysis. As expected, all tumor organoids showed MSI-H, while WT organoids did not (**Figure 2**; **Supplementary Table 8**). This is consistent with the MSI status in *de novo* tumors from *VCMsh2* mice (data not shown). Organoids derived from different tumors from the same *VCMsh2* mouse showed the similar MSI profiles (**Supplementary Table 8**). For example, 960T2A1 and 960T3D3 were derived from tumors T2 and T3, respectively, from mouse #960. Their MSI profile for three markers mU12235-A24, mL24372-A27 and mBat64 was m1 and m1, m7 and m4, and m23 and m21, respectively. Moreover, organoids generated from serially transplanted tumors *in vivo* showed higher instability compared to parental 586T2A4 and 961T3C3 organoids (**Supplementary Table 8**). For example, parental 586T2A4 had m4, m6, and m25, while P8 organoid from a serially transplanted tumor from this parental line (586T2A4-P8-T41) had m6, m11, and m37 for markers mU12235-A24, mL24372-A27 and mBat64, respectively. This indicates progressive instability during *in vivo* tumor progression.

**Figure 3:**
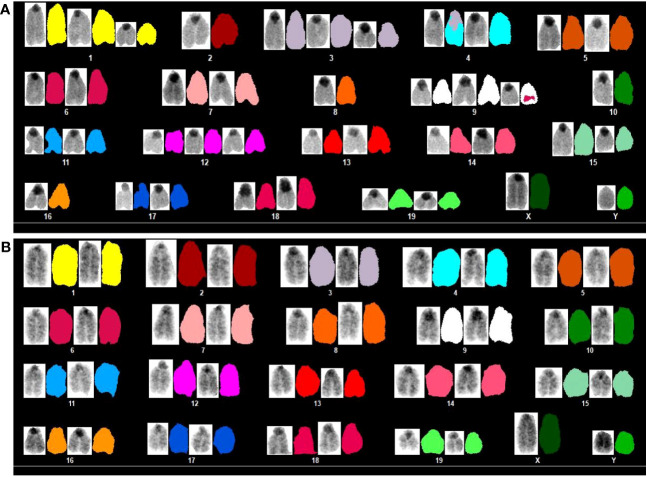
Karyotype by SKY analysis in **(A)** 586T2A4: 40,XY,+1,-2,+3,T(3;4),-8,+9(T6),-10,+12,-15,-16. T: translocation. One copy of chromosome 15 was bigger than that in **(B)** a WT normal cell.

A mixed genomic state of MSI and CIN has been reported in colorectal cancer (35, 36). To determine if CIN also occurred in *VCMsh2* MMRd tumors, tumor organoids were assessed via SKY. 586T2A4 showed high levels of chromosomal abnormality due to whole-chromosome amplification (e.g., chr1, 3, 9, and 12), deletion (e.g., chr2, 8, 10, 15, and 16), or translocation (e.g., T(3:4),+9(T6)) (**Figure 3**; **Supplementary Table 9**). However, the instability was variable and heterogenous within each organoid and among different organoid lines. For example, some cells from 586T2A4 and most cells from 357T2B2 had normal chromosomes (e.g., 40,XY for 586T2A4 and 40,XX for 357T2B2), while 546T2A2 and 961T3C3 showed high instability (e.g., amplification of chromosome 19, translocation of chromosomes 3, 4, and 6, and tetrasomy of chromosomes 1, 3, 4, 6, 8, 9, 10, 11, 17, 18, and X for 546T2A2, and loss of chromosome Y, deletion of chromosomes 13 and 18, and trisomy of chromosomes 1, 2, 3, 5, 8, 13, 14, and 17 for 961T2C3; **Supplementary Figure 5**, **Supplementary Table 9**).

### 3.3 Mutations in tumor organoids

Many recurrent FSMs have been reported in coding microsatellite repeat regions (e.g., mononucleotide repeats) in MMRd/MSI-H tumors (16, 28, 37, 38). Clinically, multiplex PCR and capillary electrophoresis (CE) (fragment analysis) and next-generation sequencing (NGS)-based assays have been approved by the Food and Drug Administration (FDA) for MSI detection. To determine whether tumor organoids from *VCMsh2* mice harbored the FSMs reported by Gebert et al. (14), targeted sequencing was performed via NGS. As expected, in all organoids, FSMs were detected with variable mutation frequency (e.g., 100% for Asxl1(-1), Xirp1(-1), and Senp6(-1), and 75% for Nacad(-1)) (**Table 1**) and high VAF, except for Maz(-1) (**Supplementary Table 10**). Consistently, FSMs were also detected in *de novo* end-stage tumors and tumor-matched mucosa tissues from *VCMsh2* mice. Interestingly, the similar mutation frequency was observed between tumors and tumor-matched mucosa, indicating that these FSMs driven by *Msh2* deletion may not be sufficient to drive the tumor development and that secondary mutations may be required.

**Table 1:** FSM frequency in organoids, and matched end-stage tumor and mucosa from *VCMsh2* mice.

Gene	Organoids (n = 10)	End-stage tumors (n = 18)	Tumor-matched mucosa (n = 16)
Asxl1(-1)	100%	94.4%	100%
Maz(-1)	20%	12.5%	7.1%
Xirp1(-1)	100%	100%	100%
Senp6(-1)	100%	94.4%	100%
Nacad(-1)	75%	94.4%	100%

-1: deletion of one nucleotide in MNR region.

Somatic mutation of *Apc* has been reported in *VCMsh2* tumors (19). Fragment analysis or Sanger sequencing for seven hotspot loci (reported by Kucherlapati et al. (19)) was performed in 43 organoid lines derived from 27 *VCMsh2* tumors (several organoid lines for some tumors). The *Apc* mutations were detected at four codons (c854, c956, c1211, and c1464), with the highest frequency at c1464 (44.4%) (**Supplementary Table 11**). The same mutation was detected in several organoids derived from the same tumor. Some organoids had more than one *Apc* mutation. For example, organoid 357T3A3 had *R956X* and *c1464 delAG*, and 979T2A2 had *c1211 delTC* and *c1464 delAG*. β-catenin is downstream of Wnt/Apc signaling. *Ctnnb1* mutation in tumor organoids was further examined via Sanger sequencing. Primers for 18 amplicons were designed to cover all the exons of *Ctnnb1*, except exon 1. Two *Ctnnb1* mutations were found in 27 organoids (7.4%): a missense mutation (*S37P*) and a coding-silent mutation (*F560*) (**Supplementary Table 12**). The same *Ctnnb1 S37P* mutation was found in five organoid lines derived from the same tumor (586T2), indicating this mutation may be an early clonal event. Interestingly, there was no *Apc* mutation in organoids from the 586T2 tumor.

Tumor suppressor p53 has been shown to play a critical role in tumorigenesis of most cancer types (39). *Trp53* mutation was assessed in 26 organoid lines from 15 tumors by targeted sequencing using 10 amplicons to cover all the coding exons of *Trp53*. Three mutations (G242ATer*, *R280C*, and *T380Q*) were detected in parental 586T2A4 and its second- and third-generation organoids, while *T380Q* was also detected in the 968T1 organoid line (**Supplementary Table 12**). *Trp53 G242D* mutation was only detected in one organoid line (961T3C3) with low VAF. Interestingly, VAF was lower in the parental line compared to organoid lines generated from serially passaged tumors (6.5% vs. 50%), indicating *in vivo* biologically relevant natural selection of pre-existing clones. Consistently, nuclear accumulation of p53 was observed in 586T2A3 and 961T3C3 organoids via IHC staining (**Figure 1A**; **Supplementary Figure 2A**). In general, *Trp53* mutation frequency was low in Msh2-deficient mouse tumor organoids. Notably, some organoids had neither *Apc*/*Ctnnb1* nor *Trp53* mutations, suggesting that other secondary mutations may drive the tumor development.

### 3.4 Frameshift peptide expression in tumor organoids

**Figure 4:**
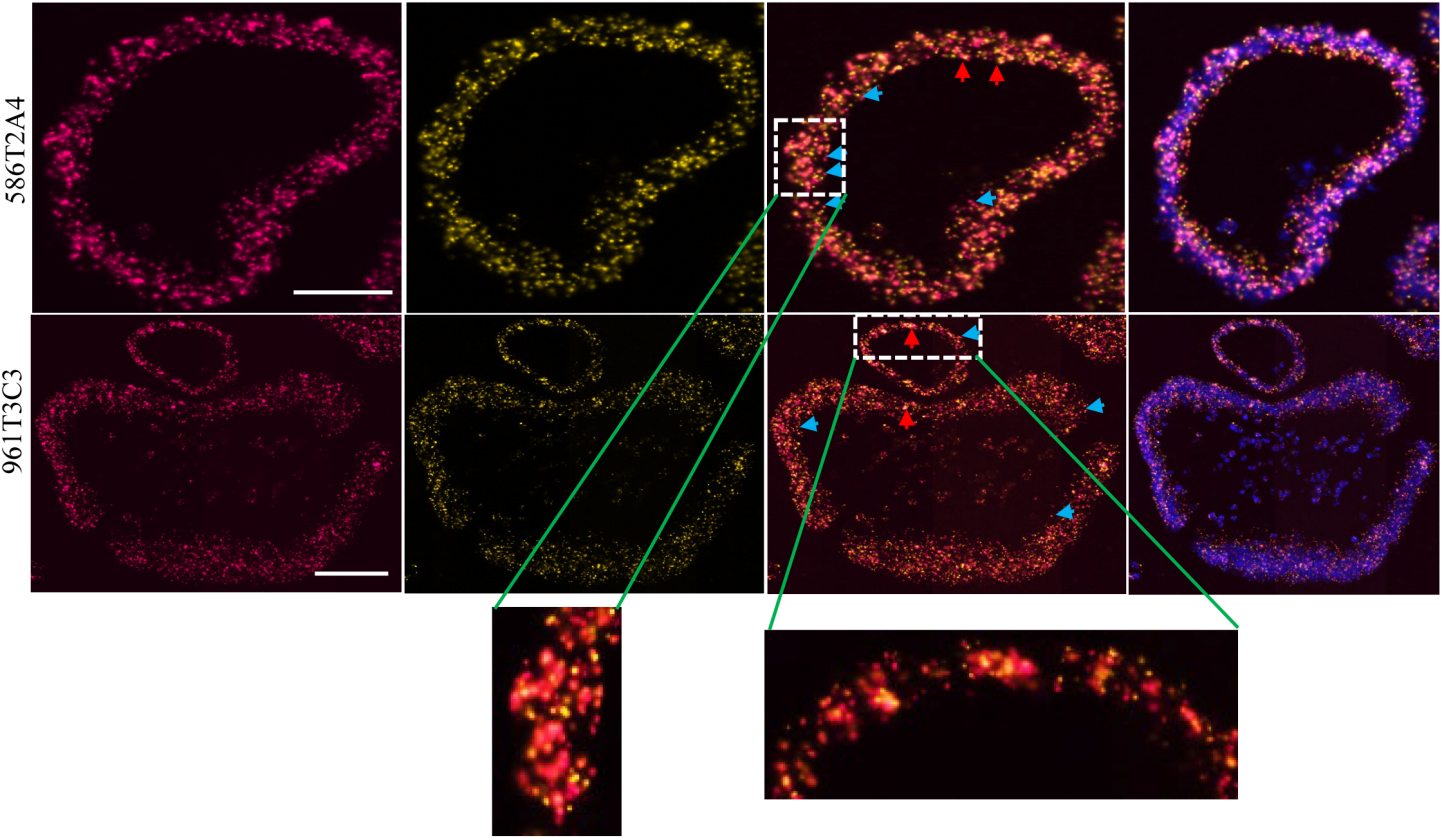
Asxl1 expression by RNA *in situ* hybridization in organoids 586T2A4 and 961T3C3. Two RNAScope® probes were designed upstream (red; left panel) and downstream (yellow; the panel next to the left) of the mononucleotide repeat region in Asxl1 mRNA. 3rd panel: merged images of two probes; right panel: merged images of two probes and DAPI for nuclei counter staining. WT: both yellow and red signals (merged as orange, red arrow). Mutant: red only (blue arrow). Scale bar: 50 µm.

FSP neoantigens have been used as vaccine targets for MMRd cancers (40). To determine whether FSPs were expressed in tumor organoids, targeted mRNA sequencing was performed via MiSeq. Frameshift-mutant Senp6 and Asxl1 mRNA were detected in both 586T2A4 and 961T3C3 organoids tested, and mutant Maz mRNA was detected only in 961T3C3 (**Supplementary Table 13**), while mutant Nacad and Xirp1 mRNA were not detectable. This may be due to poor sequencing coverage (8 and 134 reads count, respectively) or low expression of these two targets. To further confirm the expression of Asxl1 since there was no mutant-specific anti-Asxl1 antibody available, mRNA *in situ* hybridization via RNAScope® was employed. Two customized probes were designed, with one probe targeting upstream and the other targeting downstream of a mononucleotide repeat region. The sequence against the downstream probe is not present in the frameshift-mutant transcript due to the frameshift causing early termination of the transcript. Asxl1-mutant mRNA, along with WT Asxl1 mRNA, was found to be highly expressed in both tumor organoids (**Figure 4**; **Supplementary Table 14**).

### 3.5 Tumorigenicity of organoids via subcutaneous injection in syngeneic and NSG mice

**Figure 5:**
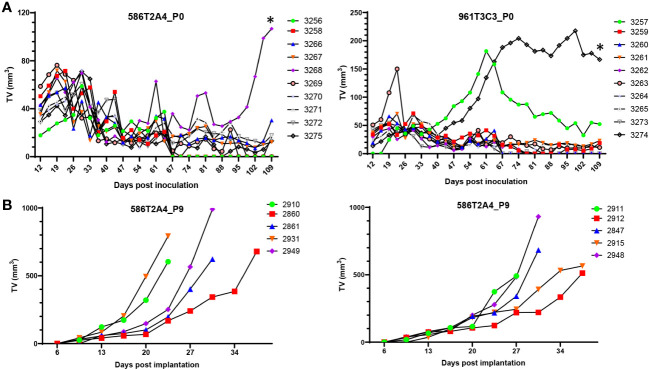
*In vivo* growth of organoids. **(A)** Growth of parental organoids 586T2A4 and 961T3C3 by s.c. injection of 1 × 10^6^ cells in C57BL/6J recipients (n = 10 per group). This was *in vivo* passage 0 (P0). *Tumor fragments from P0 tumor #3268 (left panel) and #3274 (right panel) were serially passaged s.c. *in vivo*. **(B)** Growth of 586T2A4 serially passaged tumor fragments at P9 from two different donor tumors (n = 5 per group). The median time for a tumor to reach 500 mm^3^ was 26.4 days (95% CI, 21.5 to 31.3) (left panel) and 28.3 days (95% CI, 26.4 to 35) (right panel). TV, tumor volume.

To determine the tumorigenicity of organoids derived from *VCMsh2* tumors, low-passage organoids (< P10) were injected s.c. into gender-matched syngeneic recipients with two different inocula. To our surprise, s.c. tumors did not grow well (**Figure 5A**; **Supplementary Figure 6A**). After initial growth, tumors plateaued and then, for some tumors, regressed to become non-palpable. An additional eight organoid lines were tested s.c., and a similar growth pattern was observed (data not shown). To determine whether this was due to immune editing, these organoids were injected s.c. into immune-compromised NSG mice, resulting in a similar growth pattern (**Supplementary Figure 6B**). Histological analysis showed that s.c. tumors in C57BL/6 or NSG recipients had heterogenous pathology, with adenocarcinomas mixed with cystic/mucinous features (**Supplementary Figure 7A, B**). To determine whether the regression of s.c. tumors was due to low proliferation or high apoptosis rate, Ki-67 IHC was used to assess proliferation, and TUNEL or Caspase-3 IHC was used to assess apoptosis. Results showed that these tumors had strong Ki-67 expression and a low apoptosis rate (**Supplementary Figures 7C, D**).

### 3.6 Serial *in vivo* passaging of tumor organoids

Not all s.c. tumors from organoids had growth plateau and regression. To determine whether they could grow continually without plateau or regression, tumors were serially passaged s.c. to establish a syngraft model for preclinical use. After nine passages of 586T2A4 *in vivo*, tumors grew continuously without regression (**Figure 5B**). Consistent with the parental 586T2A4 s.c. tumor histology (**Supplementary Figure 8A**, upper panel), serially passaged tumors showed classic adenocarcinoma with low mucinous features (**Supplementary Figure 8A**, middle and bottom panels). The median time for a tumor to reach 500 mm^3^ was 26.4 days (95% CI, 21.5 to 31.3; **Figure 5B**, left panel) and 28.3 days (95% CI, 26.4 to 35; **Figure 5B**, right panel). 961T3C3 was passaged to P2 *in vivo*, and the resultant tumors showed more cystic/mucinous tumor growth (**Supplementary Figure 8B**) compared to 586T2A4 (**Supplementary Figure 8A**). This is consistent with the pathology observed in *de novo* 586 and 961 tumors (**Supplementary Figure 1B**), which recapitulates different histopathological types of LS CRC tumors (41–44). P2 s.c. tumors of 961T3C3 were not further passaged *in vivo*. As shown above, Lgr5 was highly expressed in some organoids, but not others from the same organoid line (**Figure 1B**, **Supplementary Figures 2B**, **3**, **Supplementary Table 7**). To determine whether these s.c. tumor cells expressed Lgr5, we performed Lgr5 mRNA *in situ* hybridization. For 586T2A4 P0 parental s.c. tumors, strong Lgr5 expression was observed in the solid tumor mass region but not in the mucinous tumor (**Supplementary Figure 9A** left panel). Serially implanted s.c. tumors also expressed Lgr5, but cells in the inner mass had weak expression compared to those at the periphery (**Supplementary Figure 9A**, middle and right panels). Mucinous tumors had either heterogenous Lgr5 expression (546T2A4 and 577T2A4; **Supplementary Figure 9B**, left and middle panels), or no Lgr5 expression (961T3C3; **Supplementary Figure 9B**, right panel, bottom). This may be because 961T3C3 s.c. tumors were filled with thick mucus in the lumen without any live tumor cells lining the lumen (**Supplementary Figure 9B**, right upper panel).

### 3.7 An orthotopic model for MMRd/MSI-H tumors in syngeneic mice

**Figure 6:**
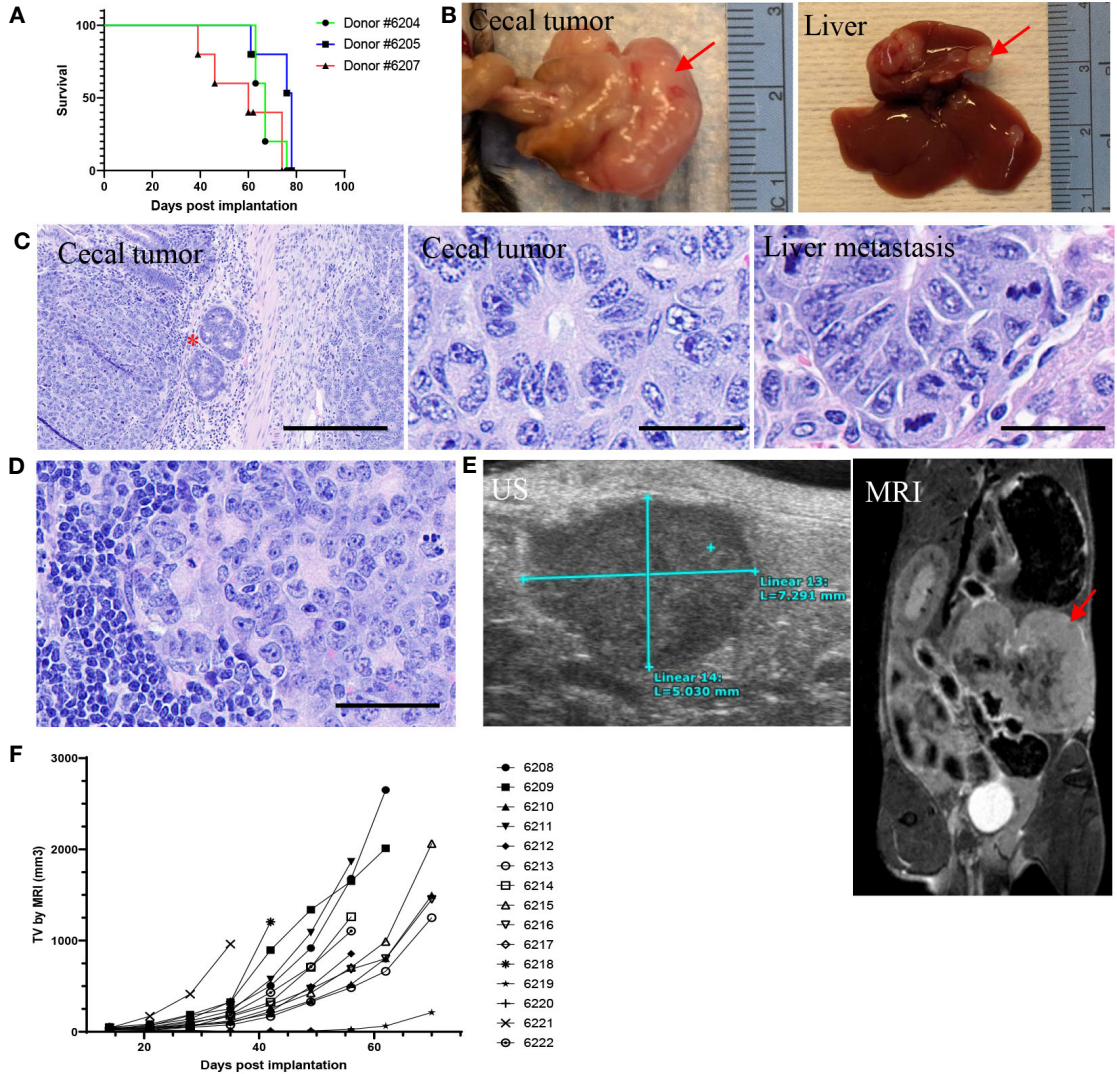
Intra-cecal implantation model developed using P8 tumor fragments from 586T2A4 organoids (n = 5 per group). **(A)** Kaplan-Meier survival curve using tumor fragments from three different donor tumors originating from 586T2A4 organoids. The median survival was 60, 67, and 78 days post implantation (dpi). **(B)** Gross primary cecal tumor and liver metastasis (red arrows). **(C)** H&E staining of cecal tumors and liver metastasis. The red star indicates the invasion of tumors into intestinal serosa. Scale bar: 200 µm (left panel) and 25 µm (middle and right panels). **(D)** H&E staining of lymph node metastasis. Scale bar: 50 µm. **(E)** Images of ultrasound (US; left) and MRI (right, red arrow points to tumor mass). **(F)** Tumor volume (TV) measured by MRI.

The impact of microenvironment on tumor growth and immune modulation has been well studied. To generate an orthotopic model and determine whether tumor cells could grow well in their native microenvironment, 2x2-mm tumor fragments were generated from three s.c. tumors derived from organoids, then implanted one fragment per animal to the wall of the cecum (n = 5 per donor). Thirteen out of 15 animals had cecal tumor growth (87% success rate; **Supplementary Table 15**) with median survival of 67, 78, and 60 days post implantation (dpi) for each donor group, respectively (**Figure 6A**). The tumors were much larger compared to *de novo* tumors (**Figures 6B, F**). Histopathological analysis showed orthotopic tumors growing on the serosal surface of the large intestine. The tumor cells formed ductal structures as adenocarcinoma with a high mitotic index (**Figure 6C**), recapitulating the *de novo* tumor pathology (**Supplementary Figure 1B**). Tumor cells also invaded the intestinal wall (**Figure 6C**) and metastasized to the liver (4/13, 31%; **Figures 6B, C**) and lymph node (4/13, 31%; **Figure 6D**), which are clinically relevant metastatic sites for LS CRC (45). Three animals had both liver and lymph node metastases. Two animals did not have cecal tumors, which might be due to surgical failure. To determine whether orthotopic tumors can be monitored by imaging, ultrasound (US) and magnetic resonance imaging (MRI) were performed. While cecal tumors were detectable by both MRI and US (**Figure 6E**), metastatic tumors could not be imaged, possibly due to the small size. A good correlation was observed between tumor volume measured by US and MRI (**Supplementary Figure 10B**).

## 4 Discussion

Organoids have been extensively used to study cancer biology and drug sensitivities (46–48). Intra- and interpatient heterogeneity in ovarian cancer (49) and heterogeneity of breast cancer subtypes (50) were investigated using organoids. A progressive association between niche-independent growth and the adenoma-carcinoma transition was observed using a comprehensive organoid library from CRC (51). A distinct mutational signature in CRC was uncovered using intestinal organoids, which reveals an underlying mutational process potentially resulting directly from past exposure to a specific type of bacteria (52). Moreover, organoids have been used in high throughput drug screening, which allows the detection of gene-drug associations (53). Although the etiology of MMRd CRC has been known for decades, it is not clear what impact thousands of FSMs throughout the genome have on tumor initiation and progression and which secondary mutations drive the tumorigenesis. It is evident that the penetrance and frequency of MMRd CRC were different among 4 MMR genes. However, it is not known whether FSMs are the same in tumors with different MMR gene deficiency. Shared frameshift neoantigens are ideal for vaccine development for MMRd cancers. Moreover, the mechanisms of resistance to immunotherapies are under-investigated in MMRd CRC. As described in this report, a panel of mouse intestine organoids from MMRd/MSI-H tumors has been generated and characterized and is well suited for mechanistic studies and *in vitro* drug screening for MMRd CRC.

Several intra-cecal syngeneic CRC models have been reported using either MSS cell lines in syngeneic mice (e.g., MC38 (54, 55) or CT26 (56–59)) or human MSI-H cell lines (e.g., HCT116) in immune-deficient mice (60, 61). Herein, we described an intra-cecal syngeneic model using tumor fragments derived from MMRd/MSI-H mice. There are several advantages of this orthotopic model over the existing ones. 1. A study cohort can be generated in a relatively short period of time compared to GEMMs (e.g., VCMsh2 model); 2. It can be used for preclinical testing in immune competent mice thus allowing the modeling of the host immune-tumor interactions, as compared to xenograft models in immunodeficient mice using cell lines derived from human patients; 3. Compared to subcutaneous models, tumors developed in this model have relevant organ site, which is required for emergence of metastasis and studying host responses and interactions with growing tumors; 4. It can be used to test site-specific dependence of therapies, assess the efficacy of therapies on metastasis, and evaluate drugs that modulate the tumor-host interaction; and 5. It can be used to study the pathogenesis of metastases for MMRd/MSI-H tumors. The implantation success rate was high (87% vs. 67% by Greenlee et al. (54) and 65% by Evans et al. (58)) and the distant metastasis was biologically relevant in this model (liver (15%) and intra-abdominal lymph node (20%) metastases in human MSI-H CRC) (45). Orthotopic CRC models have been serving as valuable tools for studying genes involved in metastasis (56) and evaluating the efficacy of immune checkpoint blockade therapies (59) and other therapies (e.g., recombinant methioninase to target the methionine (MET)-dependent cancers (62)). The model described here is the first orthotopic model of MMRd/MSI-H intestine cancer, and well suited for studying MMRd/MSI-H cancer biology and preclinically testing vaccines and immunotherapies in combination with targeted therapies for MSI-H tumors. We are currently assessing the efficacy of frameshift peptide and mRNA vaccines in this intra-cecal model.

It is evident that the microenvironment is critical for tumor growth and metastasis (63–67). In this study, we found that MMRd/MSI-H organoid s.c. tumors usually plateaued or regressed after initial growth in both syngeneic and immune-compromised mice, indicating that intrinsic factors or the microenvironment play an important role in organoid growth *in vivo*. Indeed, Li et al. reported that the tumor microenvironment can be shaped by a chemokine, CXCL1, intrinsically produced by tumor cells, which acts as a molecular “switch” (68), indicating that intrinsic factors can be responsible for the adaptability of tumor cells in the microenvironment and outcome of a therapy. Interestingly, after tumors were serially passaged s.c. *in vivo*, the organoids derived from these tumors showed good growth without regression or plateau when they were injected s.c. in syngeneic mice, suggesting that the most fit clones, presumably with driver mutations, were selected or evolved *in vivo*. Notably, distant metastasis was observed only in the intra-cecal model, not in the s.c. model or *VCMsh2* model, demonstrating the importance of the native microenvironment in metastasis. It seems that there is a requirement for further evolution of tumor cells in a more native environment for execution of a metastatic propensity during tumor progression. This was supported by evidence that the tumor-immune microenvironment of CRC cannot be modeled in s.c. models (69) and that the stroma can control tumor aggressiveness (70). In addition, past work in a melanoma model indicates that tumor location determines tissue-specific recruitment of tumor-associated macrophages (71). Thus, it is plausible to assume that tissue-specific microenvironmental factors, including the immune system, are required for tumor progression and metastasis. More research is needed to determine the factors involved in pro-metastatic tumor growth and mechanisms of immune evasion in MMRd/MSI-H tumors.

Ki-67 is expressed during all active phases of the cell cycle (G1, S, G2, and M) but absent in resting cells (G0). Thus, it has been widely used as a proliferation marker. However, Ki-67 positivity does not always correlate with proliferation. Cancer cells usually have a deregulated cell cycle and, in some cases, can be stuck at an active phase without proliferation but show positivity for Ki-67 (72, 73). It has been reported that Ki-67 was concomitantly expressed with p16, a senescence marker, in cervical epithelial cells in HPV+ women (74), indicating an abnormal cell cycle caused by HPV oncoproteins. Ki-67 positivity in s.c. tumors with plateaued growth indicates that the cell cycle of these tumor cells may be dysregulated, which can happen due to several reasons (e.g., DNA damage, mutations in certain genes related to the cell cycle, or an invoked checkpoint). To assess the proliferation status in these s.c. tumors, one can perform bromodeoxyuridine (5-bromo-2’-deoxyuridine, BrdU) labeling *in vivo*, which only incorporates into newly synthesized DNA during the S phase, and subsequently use a specific anti-BrdU antibody for detection in tumors. Interestingly, these s.c. tumors were mucinous. High mucin production may play a role in dysregulating the cell cycle and tumor growth. Past work has reported that mucins can serve as signaling molecules that alter the proliferation, differentiation, or cell-adhesion status of the tumor cells/epithelial cells (75). It remains to be determined whether blocking mucin production can increase the proliferation rate of these s.c. tumors.

The cell of origin has been studied extensively but remains elusive for most cancers. Two hypotheses have been proposed (76) and supported by experimental evidence: the stem cell origin, that stem/progenitor cells undergo tumorigenic transformation that eventually leads to tumor formation (77), and the non-stem-cell origin, that fully differentiated mature cells can undergo reprograming and dedifferentiate into progenitor-like cells with stem cell properties, which eventually give rise to tumors (78). Both stem cells and differentiated cells have been implicated as the cell of origin for CRC (77, 79–81). In this study, we found that organoids derived from Msh2-deficient tumors were of epithelial cell origin with very low expression of differentiated cell markers (e.g., Muc2, ChgA, and lysozyme). These organoids strongly expressed crypt cell markers Ephb2, Ascl2, and Klf5 but heterogeneously expressed stem cell marker Lgr5. This is consistent with the finding that about 50% of MMRd/MSI-H CRC had Lgr5 expression (82) and indicates that tumor cells were initiated either solely from Lgr5+ cells (with some of them differentiating to Lgr5− cells during tumor progression) or from both Lgr5+ stem cells and Lgr5− progenitor cells in the transit amplifying (TA) zone (since Villin-Cre is expressed in intestine epithelial cells all along the crypt-villus axis) (83). A lineage-tracing study using a multicolor Cre-reporter model showed that the Lgr5+ stem cells, only representing 5–10% of the adenoma cells, generated additional Lgr5+ cells and other adenoma cell types (84), indicating that tumors are initiated in Lgr5+ cells. However, microadenoma could be initiated in TA cells in a mouse model, although the authors concluded that it was highly unlikely that large adenomas were derived from short-lived TA cells (77). Interestingly, mice with one allele of *Msh2* deleted throughout the body and another allele specifically deleted in Lgr5+ cells (*Lgr5-CreER^T2^;Msh2^flox/-^*) survived longer (average 19 months) and had low penetrance of intestinal tumors (40%) (85) compared to *VCMsh2* mice with both alleles of *Msh2* deleted in Lgr5+ and TA cells (19) (median survival of 11.6 months, with 100% penetrance; data not shown). This strongly argues that intestinal tumors might be initiated in both Lgr5+ stem cells and Lgr5− TA cells. Thus, the cell of origin may not be stemness-dependent (although stem cells are much more efficient in tumor formation) but rather dependent on a serial event (e.g., initial *Msh2* deletion followed by accumulation of other mutations or initial *Apc* deletion followed by inflammation (86)). It remains to be determined whether deletion of *Msh2* only in intestinal TA cells can lead to tumor formation.

One of the hallmarks of cancer cells is genomic instability (87), which is usually manifested through CpG island methylator phenotype (CIMP) or variations at the nucleotide level (e.g., base pair mutation and MSI) or chromosome level (CIN) (88–90). MMRd typically leads to MSI-H phenotype. As expected, all the tumor organoids derived from *VCMsh2* tumors showed MSI-H. Interestingly, we observed increased instability in organoids derived from syngraft tumors serially passaged *in vivo* compared to parental organoids, indicating genome instability and continuous tumor evolution *in vivo*. In addition to the MSI, heterogeneous instability at the chromosome level was also detected in these organoid tumor cells. Some cells showed significant abnormality, including deletion, translocation, and amplification of whole chromosomes, while others were normal. This is consistent with the recent finding of mixed genomic states of MSI and CIN in CRC (35) and mesothelioma (31). Heterogeneity has also been reported at the genomic and transcriptomic level in LS CRC (91), which may account for the heterogenous response to immunotherapy treatment. Our data indicate that CIN is not a direct result of MMRd since all the tumor cells lacked Msh2 expression. It is not clear whether the heterogeneity of CIN is related to the MSI status in each cell or secondary mutations accumulated in these cells during tumor progression.

MMRd/MSI-H tumors usually have high mutation frequencies in microsatellite repeat regions (e.g., mono-, di- or tetra-nucleotide repeats) due to errors during DNA replication. Thus, MSI-H patients with a high mutation burden and neoantigens respond well to immunotherapy and have better survival than MSS patients. FSMs in coding mononucleotide repeat regions have been reported in MMRd/MSI-H patients and mouse models (16, 28, 37, 38, 92). The resulting FSPs can serve as neoantigens when they are expressed. Frameshift-neoantigen-based vaccines with different formulations have been tested and showed promising activities clinically and preclinically for cancer prevention and treatment (14, 15, 93–97). In this study, we confirmed that these tumor organoids and intra-cecal implanted tumors expressed characteristic FSMs/FSPs with variable mutation frequency and VAF. The significance of these FSMs/FSPs in tumor progression and immunoediting is unknown. It has been shown that FSP neoantigens can induce a T-cell response *in vitro* (98). With so many FSMs/FSPs in MMRd/MSI-H cancers, more studies are needed to assess the quality of neoantigens with high immunogenic potential for neoantigen-based vaccine development (99). Interestingly, we found that *Apc* and *Ctnnb1* mutations were mutually exclusive in organoids and some harbored *Trp53* mutation, presumably functioning as drivers for tumor development. However, some organoids had neither *Apc*/*Ctnnb1* nor *Trp53* mutations. This highlights the importance of secondary driver mutations during tumor progression (100–104) and the need to identify other secondary drivers in MMRd/MSI-H tumors.

In summary, the intra-cecal implantation model described here, driven by Msh2 deficiency, had the characteristics of MMRd/MSI-H tumors and recapitulated the *de novo* tumors developed in *VCMsh2* mice, with the potential for tumor growth monitoring by US or MRI. This is the first syngeneic model of MMRd/MSI-H intestine cancer. Expressing characteristic frameshift neoantigens in tumors enables studies to better understand the sequence and significance of FSMs and to test targeted interventions. Moreover, this model with distant metastasis allows us to study progressive genome instability and tumor evolution with heterogeneity. In addition, organoids derived from Msh2-deficient small intestine and colon tumors can be used to investigate MMRd/MSI-H cell biology and the tumorigenic process in depth (105).

## Data availability statement

The data and Python scripts used for *Trp53* mutation analysis presented here are available upon request from the corresponding author.

## Ethics statement

The animal study was approved by Animal Care and Use Committee (ACUC) at the National Cancer Institute (NCI) at Frederick. The study was conducted in accordance with the local legislation and institutional requirements.

## Author contributions

YS, SS, and RHS contributed to the concept and study design. TK, CS, LD, SSB, BS, RNB, SDM, TBY, HEL, TMP, SB, and JG contributed to the experimental planning and execution, and data acquisition, analysis, and interpretation. BK contributed to the histology analysis. BX, LW, QH, and SL contributed to the sequencing data analysis. YS drafted the manuscript. YS, SS, and RHS contributed to the critical revision of the manuscript. All authors contributed to the article and approved the submitted version.
