# Supplementary material for: Organoids and metastatic orthotopic mouse model for mismatch repair-deficient colorectal cancer
Source: Front Oncol. 2023 Sep 8;13:1223915. doi: 10.3389/fonc.2023.1223915 (PMC10516605; doi:10.3389/fonc.2023.1223915)
Supplement: Supplementary file 1 [file DataSheet_1.pdf]

Supplementary Material

1 Supplementary Materials and Methods

1.1 Tumor organoid culture medium:

- EDTA chelation buffer: 2 mM EDTA, 5.6 mmol/L Na_2HPO_4, 8.0 mmol/L KH_2PO_4, 96.2 mmol/L NaCl, 1.6 mmol/L KCl, 43.4 mmol/L sucrose, 54.9 mmol/L D-sorbitol, 0.5 mmol/L DL-dithiothreitol in distilled water
- Digestion buffer: 2.5% fetal bovine serum, 1 unit/ml of penicillin, 1 μg/ml of streptomycin, and 2.5 ng/ml of amphotericin B, 200 U/ml type IV collagenase, 125 μg/ml type II dispase in Dulbecco's Modified Eagle Medium (DMEM)
- Basal culture medium (BCM): 1 unit/ml of penicillin, 1 μg/ml of streptomycin, 2.5 ng/ml of amphotericin B, 10 mmol/L HEPES, 2 mM Glutamax, 1x N2 supplement, 1x B27 supplement, 1 mmol/L N-Acetyl Cysteine (NAC) in Advanced DMEM/F12 containing 50 ng/ml murine EGF
- Matrigel: Growth factor-reduced (GFR) and phenol red-free (Corning #356231; or VWR)

1.2 WT organoid culture medium:

Complete culture medium (CCM):

RPMI 1640 (Gibco)
10% FBS (Sigma F0926)
1% Pen/Strep (Gibco 15140)
1% L-Glutamine (Gibco 25030)
1% sodium pyruvate (Gibco 11360)
1% non-essential amino acid (Gibco 11140)
2.5% 1 M HEPES (Gibco 15630)
50 uM 2-meraptoethanol (Sigma M6250)
50 ng/mL recombinant murine EGF (Invitrogen PMG8041)
100 ng/mL recombinant murine Noggin (Peprotech 250-38)
100 ng/mL recombinant human R-Spondin (R&D Systems 4645-RS)

1.3 Induced MHC class I expression by IFNγ

MHC class I expression in organoid cells was assessed by flow cytometry analysis. Briefly, tumor and WT organoids were grown as described in the main text. Culture medium was aspirated and replaced with fresh medium containing 200 ng/ml recombinant mouse IFNγ (R&D Systems, Minneapolis, MN; Cat# 485-MI-100) and incubated at 37°C overnight (approx. 18 hours). Cells were dissociated with Dispase (Sigma D4693), then trypsinized (Thermo Fisher; Cat# 12604021) into a single cell suspension. Cells were washed and stained with either PE anti-H-2D^b, anti-H-2K^b, anti-β2-microglobulin (B2M), or the relevant isotype control (Biolegend San Diego CA; Supplementary

Table1) and recorded on a flow cytometer (BD FACScelesta). Flow data was analyzed using FlowJo Version 10.

1.4 Live animal imaging

1.4.1 Animal preparation and handling

Standard imaging and handling protocols were implemented for both imaging modalities, maintaining the rodents internal body temperature, and monitoring pulmonary respirations for administering anesthesia. The animal body temperature was maintained during anesthesia induction, imaging, and post procedure recovery using thermostat controlled (34 - 37ºC) heating pads, and imaging beds. Animals were initially anesthetized in an induction chamber using 3% isoflurane delivered at 1 liter/minute flow rate, using filtered air (0.2 µm filter) or oxygen as a carrier. Mice were then transferred to the imaging bed where a pulmonary rate of 50 - 90 breaths per minute was maintained by adjusting the isoflurane (1.5 - 2.0%). During MRI acquisitions, the pulmonary rate was monitored using the MP150 Biopac System (Biopac Systems Inc., Goleta, CA).

1.4.2 Ultrasound imaging

In preparation for imaging, to reduce the image artifacts due to the fur/air interface, anesthetized mice had the fur around the imaging site removed using animal clippers, followed by the application of a depilatory cream (SurgiCream, American International Industries, Los Angeles, CA). After removing the depilatory cream, the skin was wiped clean with a 1% acetic acid solution (pH of 2) to neutralize the high pH of the depilatory. Heated gel (Aqua-Gel, Parker Laboratory, Inc., Fairfield, NJ) was applied to match the tissue acoustic characteristics between the animal and the transducer and the acoustic focus was placed at the center of the tumor.

Ultrasound imaging was performed using the FujiFilm VisualSonics, VEVO 2100 (VisualSonics, Toronto, ON, CA). 3D tumor B-mode images were acquired using the MS-550s, 40 MHz transducer (40 and 90 µm axial and lateral spatial image resolution, respectively) with a step size of 0.076 mm. Tumor volumes were calculated using the maximum linear measurement with the ellipsoid formula ($X*Y*Z* \pi/6$). Serial imaging was performed on days 4, 6, 11, 18, 25 and 31 post fragment implantations.

1.4.3 Magnetic Resonance Imaging (MRI)

To monitor tumor progression, MRI was performed weekly on a 3.0T clinical scanner (Philips Intera Achieva, Best, The Netherlands). To achieve high throughput mice were imaged 3 at a time in a custom-built volume receive array coil for multiple mouse imaging with SENSE (SENSitivity Encoding). Mice were installed in prone position and initial survey scan was acquired in three orthogonal planes sagittal, coronal, and transverse. The survey images were used to verify mouse position and determine the imaging volume. Multislice T2 weighted turbo spin echo (T2w-TSE) sequence was applied in coronal 60 x 160 mm^2 view over a 16 mm thick slab. 32 slices with slice thickness 0.5 mm and in-plane resolution 0.18 x 0.18 mm^2 were acquired with repetition time TR 5333 ms, echo time TE 45 ms. MRI radiological DICOM images were displayed and analyzed using ITK-Snap v3.6.0 (www.itksnap.org). The adaptive paintbrush mode with an adaptive algorithm granularity and smoothness of 20 and 15, respectively, was used to segment the 3D volumes.

2 Supplementary Figures and Tables

2.1 Supplementary Figures

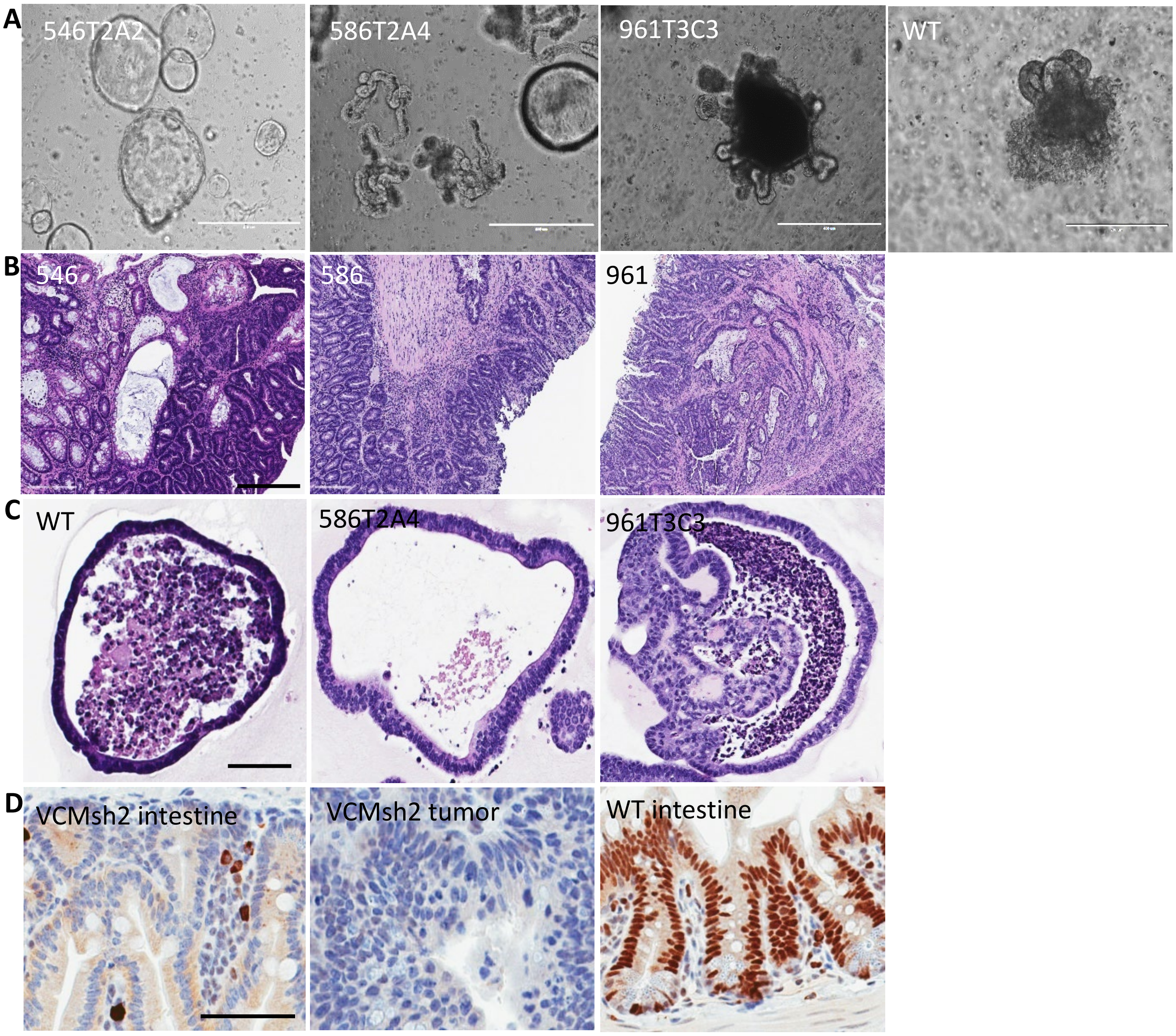

Supplementary Figure 1. (A) Morphology of three tumor organoid lines derived from *VCMsh2* tumors and a healthy organoid line from WT small intestine. Scale bar: 400 µm. **(B)** H.E. staining of *de novo* intestinal tumors from which organoids 546T2A2, 586T2A4, and 961T3C3 were derived. Scale bar: 100 µm. **(C)** H.E. staining of two tumor organoids and one WT organoid. Scale bar: 50 µm. **(D)** Msh2 IHC staining in WT small intestine, and non-tumor and tumor regions from *VCMsh2* mice. Scale bar: 50 µm.

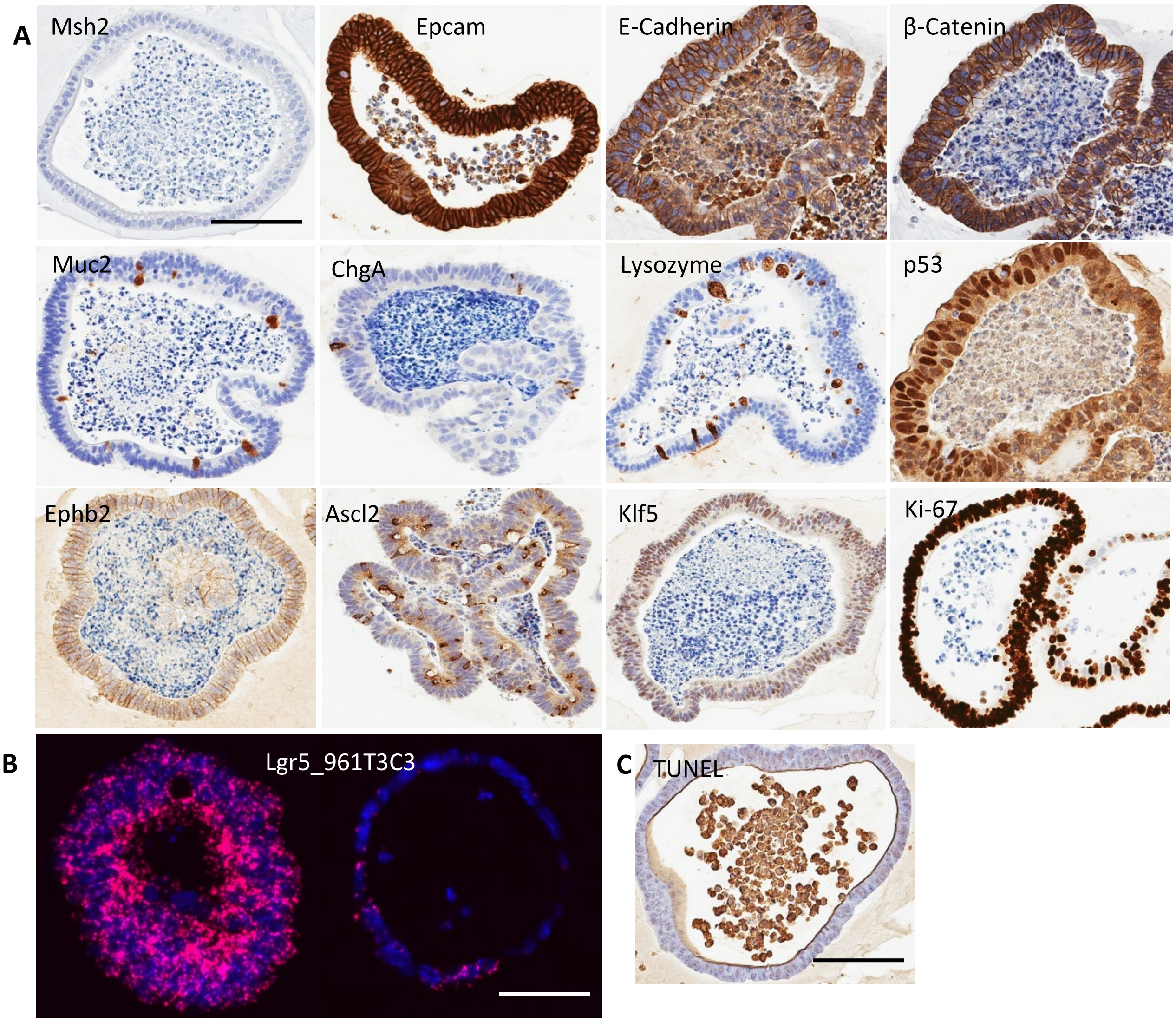

Supplementary Figure 2. Characterization of organoid line 961T3C3 by **(A)** IHC staining, **(B)** by RNA in situ hybridization via RNAscope®, and **(C)** TUNEL staining. Scale bar: 50 µm.

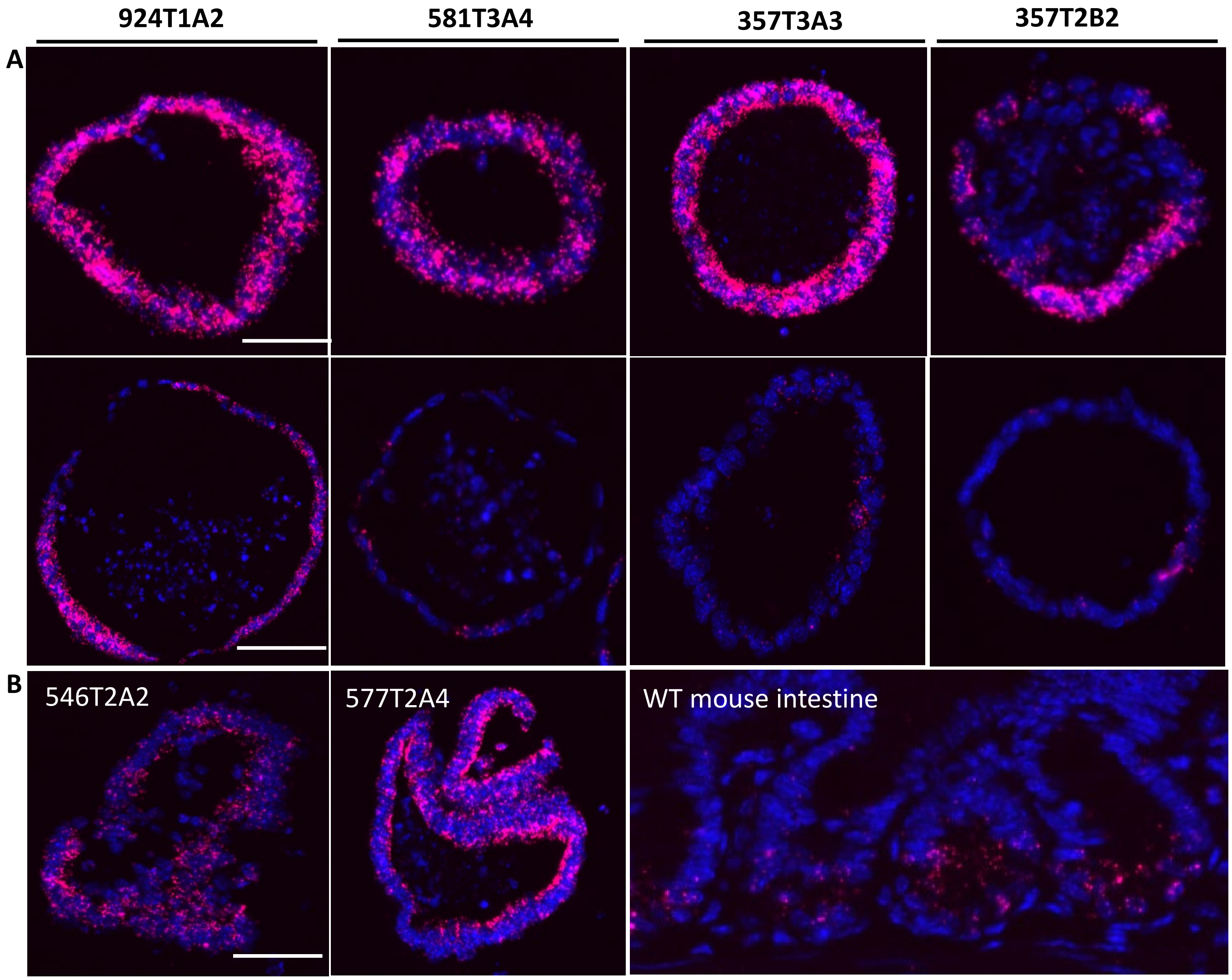

Supplementary Figure 3. RNA in situ hybridization of Lgr5 via RNAScope® in **(A)** 924T1A2, 581T3A4, 357T3A3, and 357T2B2 organoid lines derived from four different tumors, and **(B)** 546T2A2 and 577T2A4 organoid lines and WT mouse intestine. Scale bar: 50 µm.

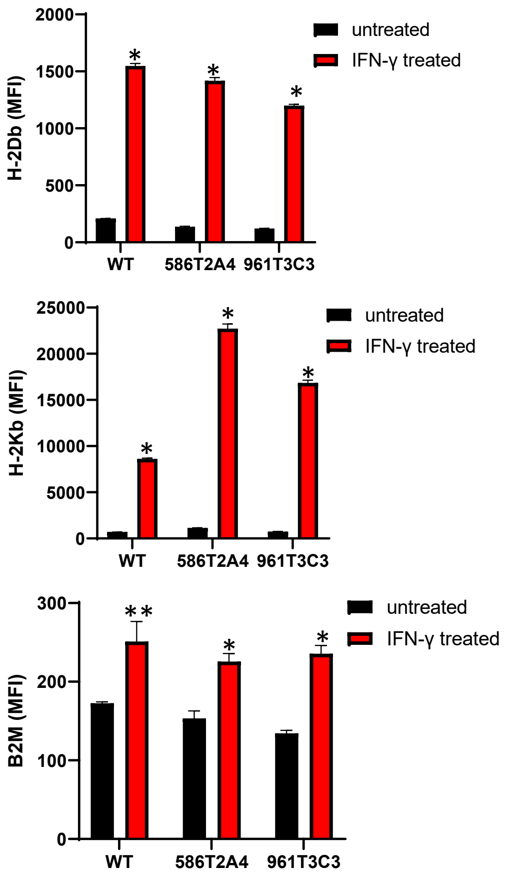

Supplementary Figure 4. Flow cytometry analysis of H-2Db, H-2Kb, and B2M expression in organoids 586T2A4 and 961T3C3 with or without IFN-γ treatment. *$p < 0.01$; **$p = 0.0187$.

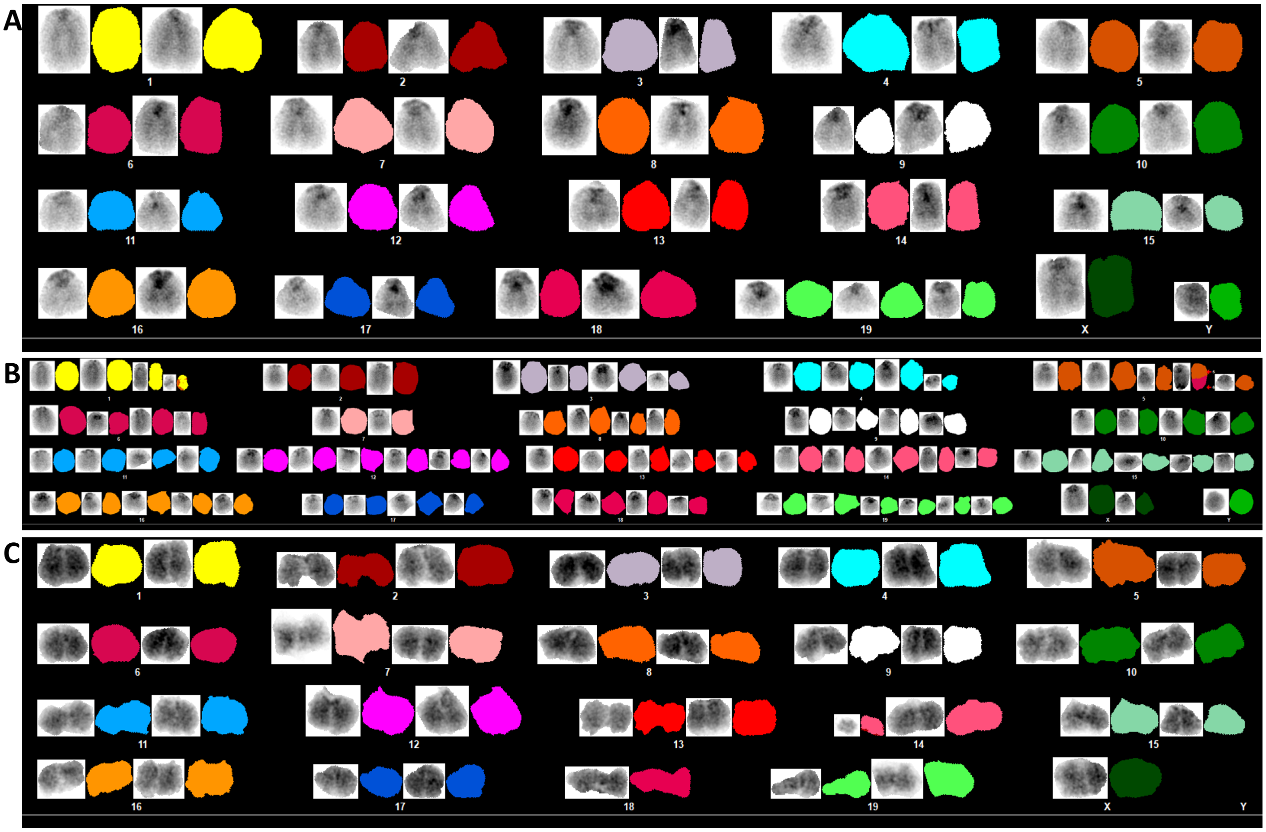

Supplementary Figure 5. Karyotype analysis of tumor organoids by SKY. **(A)** 546T2A2: 41,XY,+19; **(B)** 546T2A2: 85,XXY,+X,Tet(1),Tet(3),Tet(4),Tet(6),Tet(8),Tet(9),Tet(10),Tet(11),Tet(17),Tet(18),Ts(2),5 x5(T5;6),12 x6,13 x5,14 x5,15 x5,16 x5,19 x6; and **(C)** 961T3C3:38,XO,-Y, -14,frag(14),-18. T: translocation; Tet: tetrasomy; Ts, trisomy; The ()x the number, example (19)x6: chromosome 19, 6 copies.

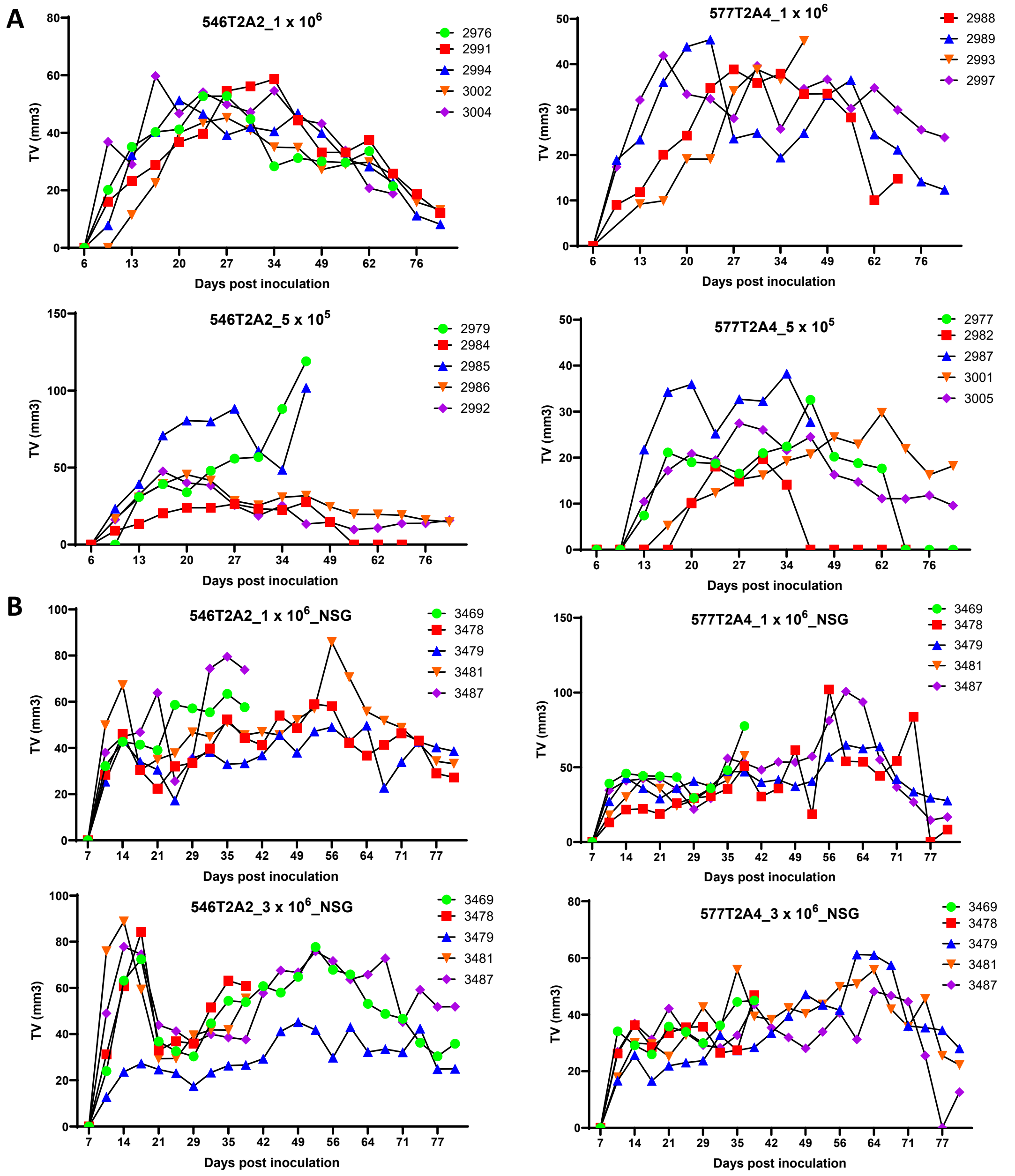

Supplementary Figure 6. *In vivo* growth of organoids 546T2A2 and 577T2A4 by s.c. injection of different number of cells in **(A)** C57BL/6J recipients and **(B)** immunodeficient NSG recipients. TV: tumor volume.

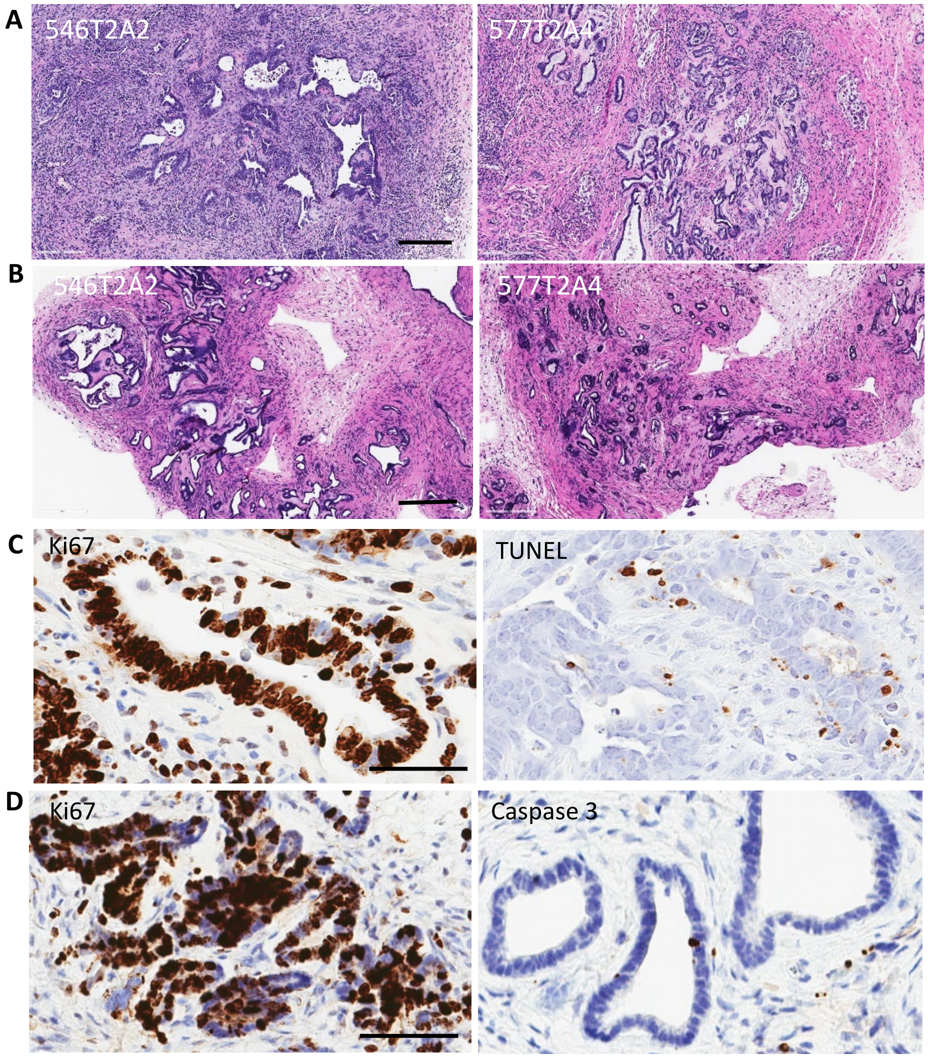

Supplementary Figure 7. H.E. staining of 546T2A2 and 577T2A4 s.c. tumors in **(A)** C57BL/6J recipients and **(B)** immunodeficient NSG recipients. Scale bar: 200 µm. **(C)** Ki-67 IHC and TUNEL staining for 546T2A2 s.c. tumor in C57BL/6J recipients. Scale bar: 50 µm. **(D)** Ki-67 and Caspase 3 IHC staining for 546T2A2 s.c. tumor in NSG recipients. Scale bar: 50 µm.

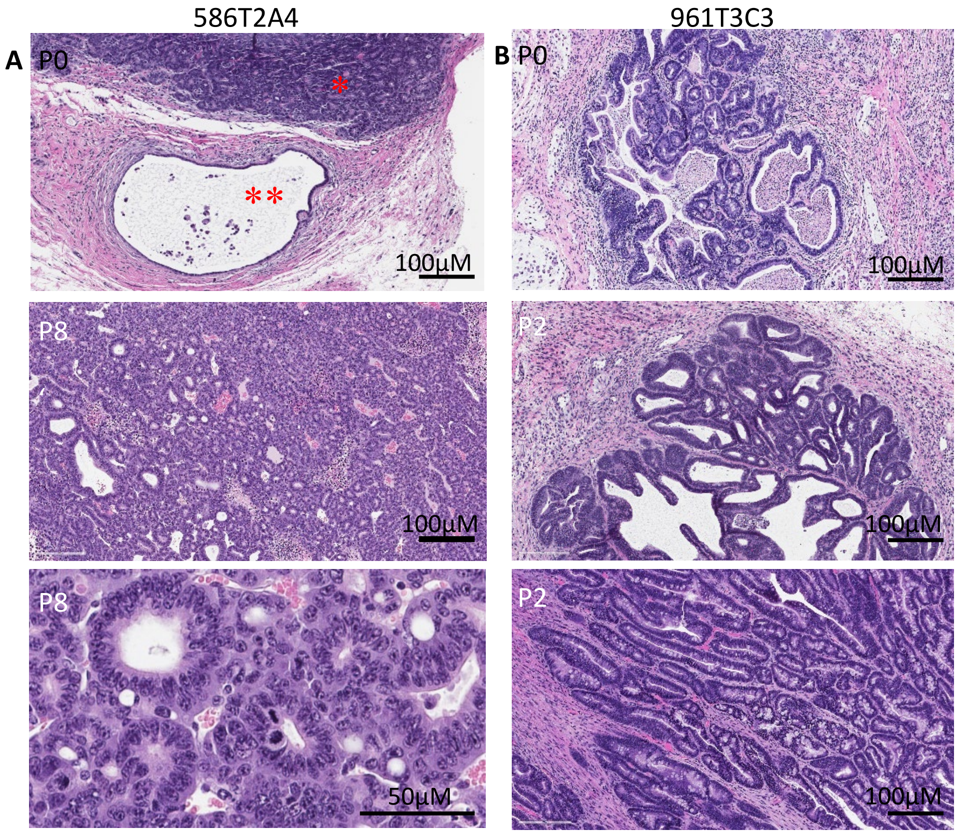

Supplementary Figure 8. H.E. images of **(A)** 586T2A4 and **(B)** 961T3C3 s.c. tumors at P0 and serially passaged tumors at P8 and P2, respectively. *Fragments generated from this solid tumor were serially passaged s.c. *in vivo*. **Mucinous tumor.

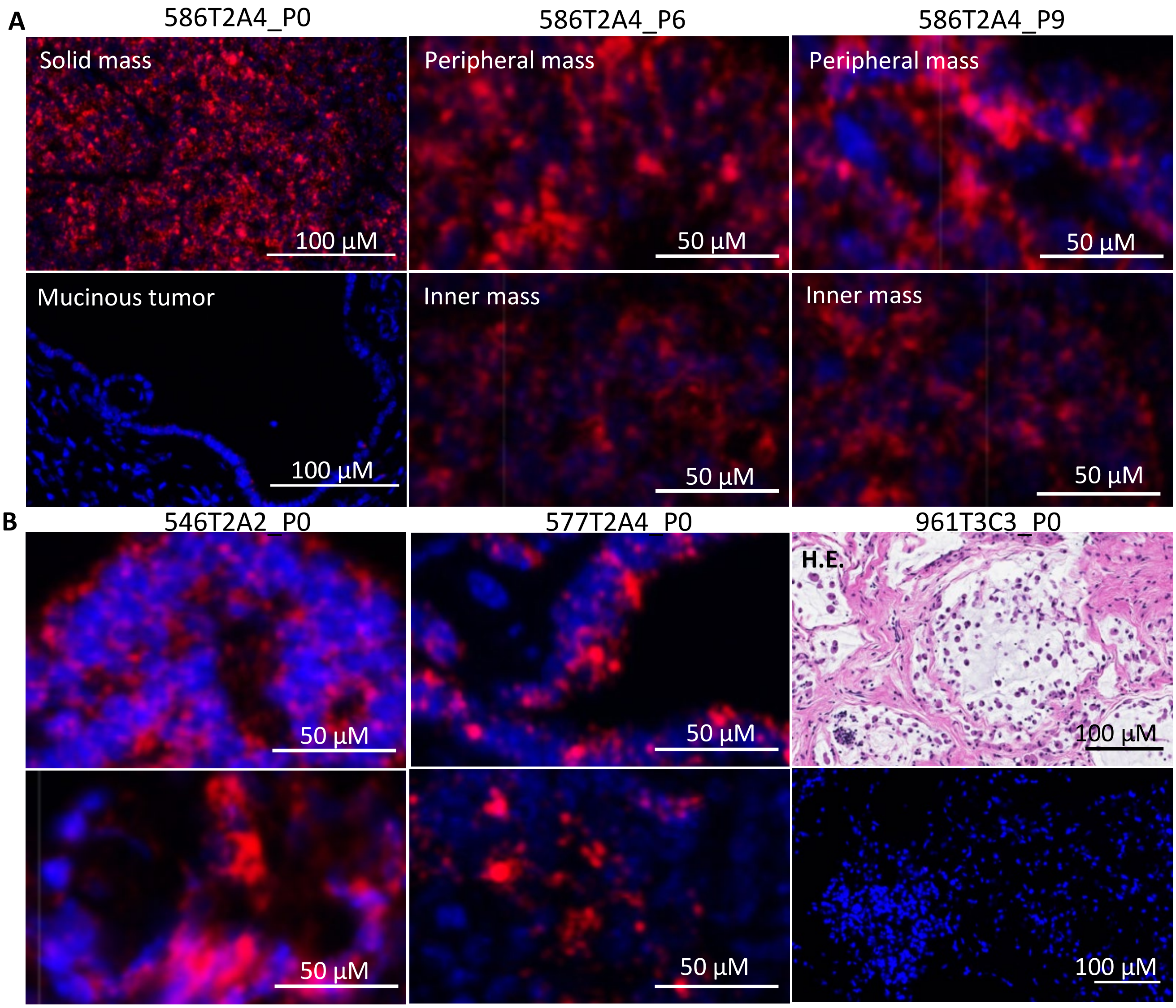

Supplementary Figure 9. RNA in situ hybridization of Lgr5 by RNAScope® in **(A)** 586T2A4 parental organoid line P0 and serially implanted s.c. tumors at P6 and P9, and **(B)** 546T2A2, 577T2A4, and 961T3C3 P0 parental lines (upper right panel: H.E. staining of 961T3C3 mucinous tumor).

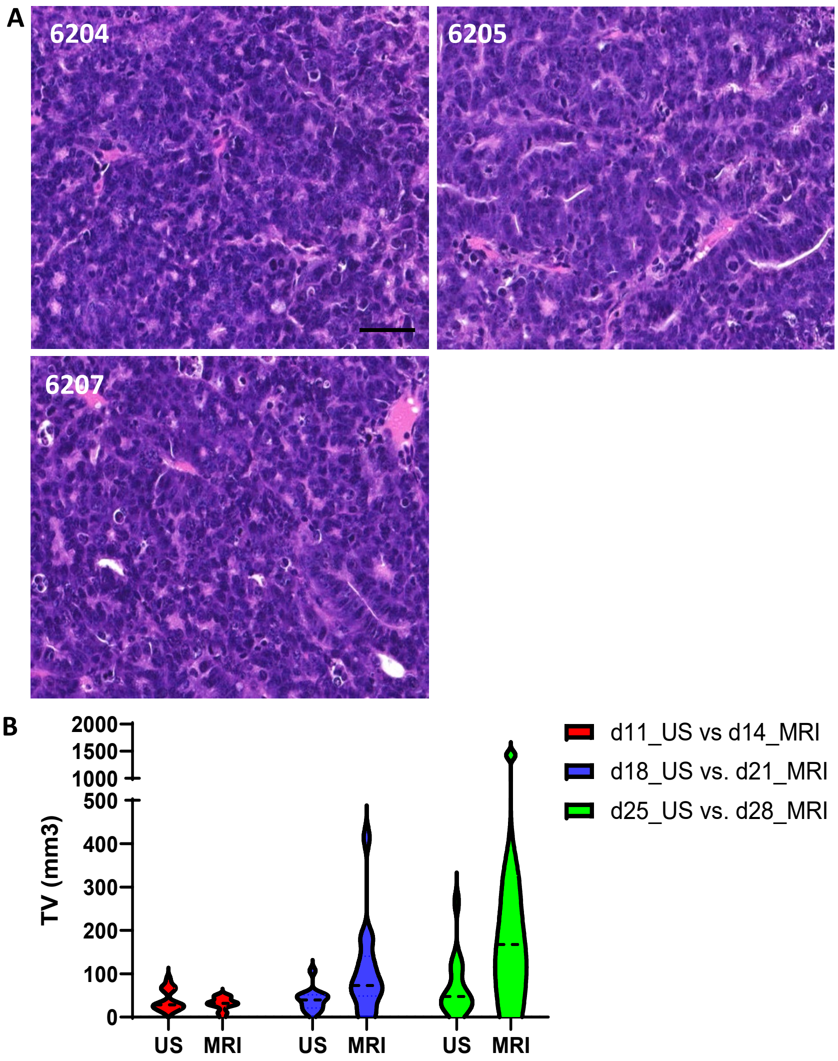

Supplementary Figure 10. Intra-cecal implantation model developed using P8 tumor fragments from 586T2A4 organoids. **(A)** H.E. images of three donor tumors used in Figure 6A. Scale bar: 50 µm. **(B)** Tumor volume (TV) measured by ultrasound (US) and MRI at different days post implantation.

2.2 Supplementary Tables

Supplementary Table 1. Antibody information.

Antibody	Vendor	Catalog #	Dilution	Application
Msh2	CST	2017S	1:800	IHC
Ki67	Cell Signaling	12202	1:200	IHC
β-catenin	Cell Signaling	8480	1:100	IHC
p53	Vector Laboratories	VP-P956	1:250	IHC
Epcam	Cell Signaling	93790	1:500	IHC
E-Cadherin	Cell Signaling	3195	1:400	IHC
Klf5	R&D Systems	AF3758	0.2 ug/ml	IHC
Muc2	Invitrogen	PA5-21329	1:4000	IHC
ChgA	Novus Biological	NB120-15160	0.33 ug/ml	IHC
Lysozyme	Abcam	ab108508	1:2500	IHC
Ephb2	R&D Systems	AF467	0.33 ug/ml	IHC
Ascl2	Novus Biological	NBP2-83940	2 ug/ml	IHC
Cleaved Caspase 3	Cell Signaling	9664	1:400	IHC
TUNEL	Millipore	S7100	N/A	IHC
PE anti-mouse H-2Db	Biolegend	111508	1:50	Flow
PE anti-mouse H-2Kb	Biolegend	1160508	1:100	Flow
PE anti-mouse B2M	Biolegend	154504	1:50	Flow
PE mouse IgG2b, κ Isotype Ctrl	Biolegend	400312	1:50	Flow
PE mouse IgG2a, κ Isotype Ctrl	Biolegend	400212	1:100	Flow
PE rat IgG2a, κ Isotype Ctrl	Biolegend	400508	1:50	Flow

Supplementary Table 2. Primers used for MSI detection by fragment analysis and *Apc* mutation by Sanger sequencing and fragment analysis.

Target	Fragment Analysis Forward Primer	Fragment Analysis Reverse Primer	Sanger Forward Primer	Sanger Reverse Primer
L24372	5'-FAM-GGGAAGACT GCTTAGGGAAGA-3'	5'-ATTTGGCTTTCAAGCATC CATA-3'	N/A	N/A
U12235	5'-FAM-GCTCATCTTC GTTCCCTGTC-3'	5'-CATTCGGTGGAAAGCT CTGA-3'	N/A	N/A
BAT64	5'-FAM-GCCCACACT CCTGAAAACAGTCAT-3'	5'-CCCTGGTGTGGCAACAT TAAGC-3'	N/A	N/A
BAT30	5'-VIC-ATTTGGCTT TCAAGCATCCATA-3'	5'-GGGAAGACTGCTTAG GGAAGA-3'	N/A	N/A
BAT37	5'-NED-TCTGCCCAA ACGTGCTTAAT-3'	5'-CCTGCCTGGGCTAA AATAGA-3'	N/A	N/A
BAT59	5'-PET-GTAATCCCTT TATTCCATTTAGCATC-3'	5'- CATCCGTAACAAGA TCTGACGT -3'	N/A	N/A
BAT67	5'-FAM-TCCATCACGTT TATATTTTAACAGAA -3'	5'-TTGCCCATTTATC ATCTAGTTCAT-3'	5'- GTAAAACGACGGCCAGT TCCATCACGTTTATATTTTAACA GAA-3'	5'- GGAAACAGCTATGACCATG TTGCCCATTTATCATCTAGTTCAT-3'
Apc c.684	5'-FAM-GCCCCTTTACTTGTTGTCAC-3'	5'-AGGCTTCCTGGTCTTTAGG-3'	5'-GTAAAACGACGGCCAGTGCCCCT TTACTTGTTGTCAC-3'	5'-GGAAACAGCTATGACCATGAGGC TTCCTGGTCTTTAGG-3'
Apc c.854	5'-FAM-ACTACGGTATTGCCCAGC-3'	5'-ACTTCTTCCATAACTTTGGC-3'	5'-GTAAAACGACGGCCAGTACTAC GGTATTGCCCAGC-3'	5'-GGAAACAGCTATGACCATGACTT CTTCCATAACTTTGGC-3'
Apc c.874	5'-FAM-AGAAAGACAGAAGTTTGGAG AG-3'	5'-ATGGAACTCGGTGGTAGA-3'	5'-GTAAAACGACGGCCAGTAGAAA GACAGAAGTTTGGAGAG-3'	5'-GGAAACAGCTATGACCATGATGG AACTCGGTGGTAGA-3'
Apc c.956	5'-FAM-AGGACATGCTCTATGCCTTA T-3'	5'-CTGTGTATCTTATGGGCTAG GT-3'	5'-GTAAAACGACGGCCAGTAGGAC ATGCTCTATGCCTTAT-3'	5'-GGAAACAGCTATGACCATGCTGT GTATCTTATGGGCTAGGT-3'
Apc c.1098	5'-FAM-GAGAATACCGATGACAAACA C-3'	5'-CGTTCACTGTAGTTGGTAG GTT-3'	5'-GTAAAACGACGGCCAGTGAGAA TACCGATGACAAACAC-3'	5'-GGAAACAGCTATGACCATGCGTT CACTGTAGTTGGTAGGTT-3'
Apc c.1211	5'-FAM-ATGTGGATCAGCCTATTGAT-3'	5'-CGCAGTAAGTCTGTATTGTT TC-3'	5'-GTAAAACGACGGCCAGTATGTG GATCAGCCTATTGAT-3'	5'-GGAAACAGCTATGACCATGCGCA GTAAGTCTGTATTGTTTC-3'
Apc c.1464	5'-FAM-CTGGGCAGACCATGCCAC-3'	5'-GTGGCGAAGTGTAACAAAG T-3'	5'-GTAAAACGACGGCCAGTCTGGG CAGACCATGCCAC-3'	5'-GGAAACAGCTATGACCATGGTGG CGAAGTGTAACAAAGT-3'

Supplementary Table 3. PCR conditions for MSI markers, *Ctnnb1*, and *Apc* mutation detection.

Targets	PCR Conditions
mBat30†<br>mBat37†	98 °C for 30 s, then 45 cycles of 98 °C for 10 s, 61 °C for 10 s, 72 °C for 15 s; followed by a final extension of 72 °C for 5 min, then holding at 4 °C
BAT64†<br>mL24372†<br>mU12235†	98 °C for 30 s, then 40 cycles of 98 °C for 10 s, 56 °C for 10 s, 72 °C for 15 s; followed by a final extension of 72 °C for 5 min, then holding at 4 °C
mBat59†	95 °C for 10 min, then 40 cycles of 95 °C for 15 s, 61 °C for 1 min; followed by a final extension of 72 °C for 7 min, then holding at 4 °C
mBat67*	98 °C for 30 s, then 40 cycles of 98 °C for 10 s, 58 °C for 10 s, 72 °C for 30 s; followed by a final extension of 72 °C for 5 min, then holding at 4 °C
Ctnnb1 Exons‡<br>Apc c.684*<br>Apc c.854†<br>Apc c.874*<br>Apc c.956*<br>Apc c.1098‡<br>Apc c.1211‡<br>Apc c.1464‡	95 °C for 10 min, then 40 cycles of 95 °C for 15 s, 60 °C for 2 min; followed by a final extension of 72 °C for 7 min, then holding at 4 °C
Apc c.854‡	98 °C for 30 s, then 35 cycles of 98 °C for 10 s, 60 °C for 10 s, 72 °C for 15 s; followed by a final extension of 72 °C for 5 min, then holding at 4 °C
Apc c.1098†<br>Apc c.1211†<br>Apc c.1464†	98 °C for 30 s, then 35 cycles of 98 °C for 10 s, 58 °C for 10 s, 72 °C for 15 s; followed by a final extension of 72 °C for 5 min, then holding at 4 °C

* Denotes the same PCR condition used for fragment analysis and Sanger sequencing.
† Denotes PCR condition used for fragment analysis only.
‡ Denotes PCR condition used for Sanger sequencing only.

Supplementary Table 4. Primers used for *Ctnnb1* mutation detection by Sanger sequencing.

Ctnnb1	**Sanger Forward Primer**	**Sanger Reverse Primer**
Exon 2	5’-GTAAAACGACGGCCAGTTG CCTACAATCTGTTTCTACTG-3’	5’-GGAAACAGCTATGACCATGGA AATACTGTTAGAAAGGCAGC-3’
Exon 3, Region 1	5’-GTAAAACGACGGCCAGTC CTTTGTTCTCTTCCCTTCT-3’	5’-GGAAACAGCTATGACCATGGG TAAATCCAACACTCACTAGG-3’
Exon 3, Region 2	5’-GTAAAACGACGGCCAGTCA AGAGCAAGTAGCTGGTAAA-3’	5’-GGAAACAGCTATGACCATGCA ACATCTGTGATGGTTCAG-3’
Exon 4, Region 1	5’-GTAAAACGACGGCCAGTCA AGAGCAAGTAGCTGGTAAA-3’	5’-GGAAACAGCTATGACCATGCC ACCTAGAATAAAGGATGAAG-3’
Exon 4, Region 2	5’-GTAAAACGACGGCCAGTTGA TTAACTATCAGGATGACGC-3’	5’-GGAAACAGCTATGACCATGTA CAACGAGCTGTCTCTACATC-3’
Exon 5, Region 1	5’-GTAAAACGACGGCCAGTACC AGGTAAGCAGTGATGTAAC-3’	5’-GGAAACAGCTATGACCATGCA GTCTGTTGTAATAGCCAAGA-3’
Exon 5, Region 2	5’-GTAAAACGACGGCCAGTAAT GATGTAGAGACAGCTCGTT-3’	5’-GGAAACAGCTATGACCATGAC TTTCATCACTCTACCTTGCT-3’
Exon 6	5’-GTAAAACGACGGCCAGTAAT GATGTAGAGACAGCTCGTT-3’	5’-GGAAACAGCTATGACCATGAA ACACCATGTCCTCTGTCTAT-3’
Exon 7	5’-GTAAAACGACGGCCAGTATT GTAGCACAGGAGTCACATA-3’	5’-GGAAACAGCTATGACCATGAA CCACTTCTCTGGTACTAAGC-3’
Exon 8	5’-GTAAAACGACGGCCAGTGTT ATCACTGGCAAGGTAGATT-3’	5’-GGAAACAGCTATGACCATGCA CAGGTGACCACATTTATATC-3’
Exon 9, Region 1	5’-GTAAAACGACGGCCAGTAAA ACTGTCTTTGGACTCTCAG-3’	5’-GGAAACAGCTATGACCATGAG TAGACAACACAACAAAGCAC-3’
Exon 9, Region 2	5’-GTAAAACGACGGCCAGTAG GGAAGACATCACTGAGC-3’	5’-GGAAACAGCTATGACCATGGT TGCCTACAGAGGAAAGAAT-3’
Exon 10	5’-GTAAAACGACGGCCAGTAT CTGTGCTCTTCGTCATCT-3’	5’-GGAAACAGCTATGACCATGCA GTACATTACACTCCATGTCC-3’
Exon 11	5’-GTAAAACGACGGCCAGTTA GGAGCCTTCCGTTATACTC-3’	5’-GGAAACAGCTATGACCATGGA GAAAACACAGAAACAAGACC-3’
Exon 12	5’-GTAAAACGACGGCCAGTACT TGAGTCCCTAGACATTGAG-3’	5’-GGAAACAGCTATGACCATGTT CTTGTAATCCTGTGGCTT-3’
Exon 13	5’-GTAAAACGACGGCCAGTCTC CATAGTTGCTTTATTCTCC-3’	5’-GGAAACAGCTATGACCATGAA ACCAACTCTCAACTAACTGC-3’
Exon 14	5’-GTAAAACGACGGCCAGTCA TCTGTCAGTGTTTATTCCTG-3’	5’-GGAAACAGCTATGACCATGCT ATGAATCACCCATCCTAGAC-3’
Exon 15	5’-GTAAAACGACGGCCAGTTGT GTGGTAGATAGTCTCAGGA-3’	5’-GGAAACAGCTATGACCATGCA AGGTATGTATCTGTTTGGTG-3’

Supplementary Table 5. Sequencing primers for *Trp53*.

Target	Forward Primer	Reverse Primer
Trp53 Exon 2	5'-GTAAAACGACGGCCAGTATT CTACCCTTTCCTATAAGCC-3'	5'-GGAAACAGCTATGACCATGCC ATACCATGTTTGAACACTAC-3'
Trp53 Exon 3	5'-GTAAAACGACGGCCAGTCAT TGACTACATAGCAAGTTGG-3'	5'-GGAAACAGCTATGACCATGCA AGTCCCTTTCTGCTCTGG-3'
Trp53 Exon 4	5'-GTAAAACGACGGCCAGTAC ACAGTCCTGAGGGTTCTTC-3'	5'-GGAAACAGCTATGACCATGAA AGGTCACACGAAAGACAACTC-3'
Trp53 Exon 5	5'-GTAAAACGACGGCCAGTTA GTTCCCCACCTTGACAC-3'	5'-GGAAACAGCTATGACCATGC CACAGGCGGTGTTGAG-3'
Trp53 Exon 6	5'-GTAAAACGACGGCCAGTGG CTTCTGACTTATTCTTGCTC-3'	5'-GGAAACAGCTATGACCATGC AGCTTGCACCTCTAAGC-3'
Trp53 Exon 7	5'-GTAAAACGACGGCCAGTGG TGGAATATCCCTACTCTACA-3'	5'-GGAAACAGCTATGACCATGG AAACAGGCTAACCTAACCTAC-3'
Trp53 Exon 8	5'-GTAAAACGACGGCCAGTCCT AGTTTACACACAGTCAGGA-3'	5'-GGAAACAGCTATGACCATGCT CCGCCTCCTTGGT-3'
Trp53 Exon 9	5'-GTAAAACGACGGCCAGTT CACCTCTTGCTCTCTCCT-3'	5'-GGAAACAGCTATGACCATGC AAGCTAATGTCACGGCTAGA-3'
Trp53 Exon 10	5'-GTAAAACGACGGCCAGTCA AAAACCTGTAAGTGGAGC-3'	5'-GGAAACAGCTATGACCATGA GGTCTGGGTAGAGCACC-3'
Trp53 Exon 11	5'-GTAAAACGACGGCCAGTAG CCCAAACTGCTAGCTC-3'	5'-GGAAACAGCTATGACCATGAG TCATAAGACAGCAAGGAGAG-3'

Supplementary Table 6. Summary of organoids generated.

	Small intestine tumor organoids	Colon tumor organoids
Total # of VCMsh2 mice used for organoid generation	27	5
# of VCMsh2 mice with organoids successfully established	16	5
Total # of tumors used for organoid generation	102	6
# of tumors with organoids successfully generated	28	5
# of Organoid lines attempted*	611	51
Established organoid lines (passaged up to P3 in vitro)	125	29
Success rate at the animal level	59%	100%
Success rate at the tumor level	27%	83%
Success rate at the organoid line level**	20%	57%

Supplementary Table 7. Summary of marker staining quantification in Figure 1.

Markers	% Positive cells from total cells (Mean)	SEM
Msh2	0	0
Epcam	100	0
E-cadherin	99.13	0.53
β-catenin	99.15	0.39
Muc2	0	0
ChgA	0.25	0.25
Lysozyme	0	0
p53	93.85	3.4
Ephb2	91.89	4.06
Ascl2	36.18	5.62
Klf5	81.01	4.31
Ki67	95.65	1.01
TUNEL	9.86	3.98
Lgr5 in high expressing organoids from the same organoid line	95.67	1.03
Lgr5 in low expressing organoids from the same organoid line	8.7	2.07

Four areas on the stained section from each organoid line were quantified using HALO® image analysis platform (indica labs, Albuquerque, NM). Each area had up to 10 organoids. Data is expressed as mean of % positive cells out of total cells in these four areas. Standard Error of the Mean (SEM) was calculated by dividing the standard deviation by the square root of four.

Supplementary Table 8. Summary of MSI status in organoids assessed by fragment analysis.

Organoids ID[&]	mU12235-A24	mL24372-A27	mBat64	mBat30	mBat37	mBat59	mBat67
334T5B4	m2	m5	m29				
357T3A4	m5	m6	m19				
530T2A3	m3	m7	m25				
546T2A2	m6	m5	m20				
546T2A3	m4	m5	m19				
577T2A4	m1	m5	m24				
577T2D4	m1	m4	m20				
577T4D2	m2	m4	m21				
581T3B3	m4	m7	m21				
581T5C1	m3	m4	m19				
582T1A6	m2	m2	m14				
924T1A4	m4	m4	m17				
960T2A1	m1	m7	m23				
960T3D3	m1	m4	m21				
968T1C2	m5	m7	m17				
979T3D4	m2	m3	m24				
961T3D4	m5	m1 and m9	m26				
961T3C3	m6	m2 and m11	m30				
961T3C3-P2-T1*	m3 and m8	m4 and m11	m38				
586T2A4	m4	m6	m25				
586T2A4-P2-T1*	m4	m8	m35				
586T2A4-P2-T2*	m6	m11	m32				
586T2A4-P2-T3*	m6	m9	m32				
586T2A4-P8-T11**	m6	m11	m36	m11	m10	m28	m26
586T2A4-P8-T21**	m6	m11	m37	m11	m10	m27	m26
586T2A4-P8-T31**	m7	m11	m37	m11	m10	m27	m25
586T2A4-P8-T41**	m6	m11	m37	m12	m11	m27	m26
WT organoids	wt	wt	wt	wt	wt	wt	wt

[&]The three numerical number indicates mouse ID. T: tumor ID. For example, 586T2A4 was from animal #586 tumor #2 and this organoid line was A4. Organoids with the same mouse ID indicates that they were generated from the same mouse. Organoids with the same mouse ID and tumor ID indicates that they were derived from the same tumor (e.g., 546T2A2 and 546T2A3) or with different tumor ID (e.g., 581T3B3 and 581T5C1) indicates they were from the same animal, but different tumors. 586T2A4 and 961T3C3 organoids were serially passaged s.c. and new organoids were generated from passage 2 (P2) and 8 (P8) s.c. tumors.
*Derived from P2 serially passaged tumors
**Derived from P8 serially passaged tumors
wt: means that the fragment size was the same as that from tail DNA. m: minus.
Blank wells: not tested

Supplementary Table 9. Summary of SKY analysis from five tumor organoids.

Organoid ID	Cell ID	Results
586T2A4; male	1	22
	2	40,XY,+Del(1),-2,-8,+Del(9),-10,+12
	3	34
	4	41,XYY,+4,+8,-12,-13
	5	40,XY
	6	25,T(4;15)
	7	40,XY
	8	40,XY
	9	40,XY split chromatids but looks normal
	10	40,XY,+1,-2, +3, T(3;4),-8,+9(T6),-10,+12,-15,-16
	11	35
961T3C3; male	1	39,XO
	2	39,XO
	3	39,XO, Del(13)
	4	66,XX,Ts(1),Ts(2),Ts(3),Ts(5),Tet(6),Tet(7),Ts(8),Tet(9),Tet(10),Tet(11),Tet(12),Ts(13),Ts(14),Tet(15),Ts(17),Tet(18),Tet(19)
	5	39,XO,Del(13)
	6	39,XO, Break at 16B
	7	39,XO, break
	8	39,XO
	9	38,XO,-Y,-14,frag(14),-18
	10	39,XO
357T2B2; female	1	40,XX,+12,+12,-14,-16
	2	40,XX
	3	40,XX
	4	40,XX
	5	80,XXXX
	6	40,XX
	7	40,XX
	8	40,XX
	9	40,XX
	10	40,XX
	11	40,XX
	12	40,XX
577T2A4; male	1	39, X,-Y
	2	40,XY(4)
	3	>80
	4	41,XY,+19
546T2A2; male	1	41,XY,+19
	2	85,XXY,+X,Tet(1),Tet(3),Tet(4),Tet(6),Tet(8),Tet(9),Tet(10),Tet(11),Tet(17),Tet(18),Ts(2),5x5(T5;6),12x6,13x5,14x5,15x5,16x5,19x6
	3	40 XY,+1,-2,+3,T(3,4),-8,+9(T6),-10,+12,-15,-16,+M

Up to 3-12 cells per organoid were analyzed. T: translocation; Tet: tetrasomy; Ts, trisomy; The ()x the number, example (19)x6: chromosome 19, 6 copies.

Supplementary Table 10. Variant allele frequency (VAF) of five genes in tumor organoids and WT organoids.

Gene*	586T2A4	586T2A4-P2-T1	586T2A4-P2-T2	586T2A4-P2-T3	586T2A4-P8-T11	586T2A4-P8-T21	586T2A4-P8-T31	586T2A4-P8-T41	961T3C3	WT organoid
Asxl1 (-1)	0.528	0.741	0.533	0.539	0.546	0.542	0.554	0.576	0.546	0.020
Maz (-1)	0.082	0.034	0.008	0.013	0.040	0.0267	0.057	0.008	0.024	0.031
Nacad (-1)	0.564	0.5	0.849	0.861	0.566	0.732	0.735	0.455	0.641	0.523
Senp6 (-1)	0.558	0.566	0.564	0.575	0.255	0.268	0.256	0.254	0.278	0.191
Xirp1 (-1)	0.546	0.548	0.528	0.541	0.517	0.550	0.533	0.557	0.605	0.067

*-1: one nucleotide deletion in the MNR region of our interest.

Supplementary Table 11. Hotspot *Apc* mutations in *VCMsh2* tumor organoids (n=27 tumors).

Apc Codon	Mutation	Consequence	WT sequence&	# of organoid lines with mutations
684*	wt	n.a.	n.a.	0
874*	wt	n.a.	n.a.	0
854*	C to T	Arg to Stop	AGA GAG **C**GA GGT	3
956*	C to T	Arg to Stop	TAT AAA **C**GA TCT	1
1098**	wt	n.a.	n.a.	0
1211**	del TC	Frameshift	CAT CTC TC**T C**CA AGC	4
1464**	del AG	Frameshift	AGA GAG **AG**T GGG	12

&Bolded base pairs have mutations described in the second column.
Analysis was done by *Sanger sequencing or **fragment analysis.

Supplementary Table 12. *Trp53* and *Ctnnb1* mutations in *VCMsh2* tumor organoids.

***Trp53* by MiSeq*(Organoids from 15 tumors)**	
*G242ATer**	Parental organoids from 586T2 tumor and organoids from serially passaged tumors
G242D	961T3C3
R280C	Parental organoids from 586T2 tumor and organoids from serially passaged tumors
T380Q, frameshift	Organoids from 968T1 and 586T2 tumors
***Ctnnb1* by Sanger** (Organoids from 27 tumors)**	
S37P	Parental organoids from 586T2 tumor and organoids from serially passaged tumors
F560 (coding silent)	Organoids from 586T1 tumor

*10 amplicons; **18 amplicons

Supplementary Table 13. Mutant mRNA sequencing of five genes in two tumor organoids.

Mutant mRNA Seq*	586T2A4 (p value)	961T3C3 (p value)	VAF_ median	background_ VAF_ median	Coverage_ median
Senp6 (-1)	0	0	0.305	0.227	217826
Asxl1 (-1)	0	0	0.275	0.019	13553
Maz (-1)	0.07561	0.004467	0.024	0.02	47544
Xirp1 (-1)	0.297523	0.536152	0.109	0.133	134
Nacad (-1)	NA	NA	0.646	0.385	8

*-1: one nucleotide deletion in the MNR region of our interest.

Supplementary Table 14. Summary of WT and mutant Asxl1expression in Figure 4.

	A: % Cells with 5' probe positive only	B: % Cells with 3' probe positive only	Ratio of A/B
**Tumor organoid (n=14)**	15.4 ± 0.99	3.99 ± 0.34	4.44 ± 0.48
**WT organoid (n=1)**	4.46	2.79	0.63

Supplementary Table 15. Tumor engraftments following intra-cecal implantation of tumor fragment.

	Count	%
Surgery survival	15/15	100%
One month survival	15/15	100%
Successful tumor inoculation	13/15	87%
Tumors with organ metastases	5/13	38%
Liver metastases	4/13	31%
Lymph node metastases	4/13	31%
